# Gene network and pathway analysis of bovine mammary tissue challenged with *Streptococcus uberis *reveals induction of cell proliferation and inhibition of PPARγ signaling as potential mechanism for the negative relationships between immune response and lipid metabolism

**DOI:** 10.1186/1471-2164-10-542

**Published:** 2009-11-19

**Authors:** Kasey M Moyes, James K Drackley, Dawn E Morin, Massimo Bionaz, Sandra L Rodriguez-Zas, Robin E Everts, Harris A Lewin, Juan J Loor

**Affiliations:** 1 Department of Animal Sciences, University of Illinois, 1207 West Gregory Drive, Urbana, 61801, USA; 2 College of Veterinary Medicine, University of Illinois, 2001 South Lincoln Avenue, Urbana, 61802, USA; 3 Institute for Genomic Biology, University of Illinois, 1206 West Gregory Drive, Urbana, 61801, USA; 4 Aarhus University, Faculty of Agricultural Sciences, Research Centre Foulum, PO Box 50, DK-8830 Tjele, Denmark; 5 Sequenom, Inc, 3595 John Hopkins Court, San Diego, CA 92121, USA

## Abstract

**Background:**

Information generated via microarrays might uncover interactions between the mammary gland and *Streptococcus uberis *(***S. uberis***) that could help identify control measures for the prevention and spread of *S. uberis *mastitis, as well as improve overall animal health and welfare, and decrease economic losses to dairy farmers. The main objective of this study was to determine the most affected gene networks and pathways in mammary tissue in response to an intramammary infection (**IMI**) with *S. uberis *and relate these with other physiological measurements associated with immune and/or metabolic responses to mastitis challenge with *S. uberis *O140J.

**Results:**

*Streptococcus uberis *IMI resulted in 2,102 (1,939 annotated) differentially expressed genes (**DEG**). Within this set of DEG, we uncovered 20 significantly enriched canonical pathways (with 20 to 61 genes each), the majority of which were signaling pathways. Among the most inhibited were *LXR/RXR Signaling *and *PPARα/RXRα Signaling*. Pathways activated by IMI were *IL-10 Signaling *and *IL-6 Signaling *which likely reflected counter mechanisms of mammary tissue to respond to infection. Of the 2,102 DEG, 1,082 were up-regulated during IMI and were primarily involved with the immune response, e.g., *IL6*, *TNF*, *IL8, IL10, SELL, LYZ*, and *SAA3*. Genes down-regulated (1,020) included those associated with milk fat synthesis, e.g., *LPIN1, LPL, CD36*, and *BTN1A1*. Network analysis of DEG indicated that *TNF *had positive relationships with genes involved with immune system function (e.g., *CD14, IL8, IL1B*, and *TLR2*) and negative relationships with genes involved with lipid metabolism (e.g., *GPAM*, *SCD*, *FABP4*, *CD36*, and *LPL*) and antioxidant activity (*SOD1*).

**Conclusion:**

Results provided novel information into the early signaling and metabolic pathways in mammary tissue that are associated with the innate immune response to *S. uberis *infection. Our study indicated that IMI challenge with *S. uberis *(strain O140J) elicited a strong transcriptomic response, leading to potent activation of pro-inflammatory pathways that were associated with a marked inhibition of lipid synthesis, stress-activated kinase signaling cascades, and PPAR signaling (most likely PPARγ). This latter effect may provide a mechanistic explanation for the inverse relationship between immune response and milk fat synthesis.

## Background

Mastitis is one of the most costly of all metabolic diseases and disorders in the dairy industry and occurs most frequently during early lactation [1,2]. The innate immune response, primarily consisting of milk macrophages and neutrophils (**PMN**), is the first line of defense against invading pathogens. The initial stages associated with innate immunity in the mammary gland are not well understood. Mammary epithelial cells (**MEC**) have immunological functions that contribute to the initial response to an intramammary infection (**IMI**) [3]. Researchers have used MEC lines or mammary tissue biopsies to study the immunological role of MEC through response to in vitro challenges with both Gram-positive and Gram-negative bacteria [4,5] Microarray as well as quantitative reverse transcription-PCR (**qPCR**) technology could provide useful information on additional signals produced by MEC during an IMI [6-8].

*Streptococcus uberis *(***S. uberis***) is a major environmental mastitis-causing pathogen [9]. Infections due to *S. uberis *are predominantly subclinical (ca. 95%) and are responsible for up to 16% and 33% of clinical cases per year in the United States and the United Kingdom [10,11]. Subclinical mastitis is the dominant form of mastitis affecting cows and frequently goes undetected and untreated for extended periods of time, which can result in spreading to other cows and significant reductions in profitability due to losses of production and milk premiums [1]. Recently, Swanson et al. [8] examined the mammary tissue transcriptome via microarray technology of 5 Friesian heifers in mid-to-late lactation after IMI with a noncapsular strain of *S. uberis *(Strain 233). Genes involved with immune response were up-regulated and genes involved in lipid metabolism and cell death were down-regulated after IMI with *S. uberis*. The capsular strain O140J has been shown to be more resistant to PMN phagocytosis and more capable of establishing infection when compared to a noncapsular strain [12,13]. However, the pathogenic mechanisms of *S. uberis *strain O140J are still unclear, thus, transcriptomic evaluation of mammary tissue gene expression after IMI with *S. uberis *O140J is clearly warranted. More importantly, identifying molecular pathways and gene networks affected by IMI with this strain would yield mechanistic information of the underlying tissue adaptations to infection.

The main objective of this study was to determine the most affected gene networks and pathways in mammary tissue in response to an IMI with *S. uberis *O140J. We hypothesized that IMI with *S. uberis *would up-regulate genes involved with immune response and alter expression of genes involved with milk synthesis and composition as well as tissue function.

## Results

### Indicators of clinical response to IMI challenge

All cows developed both local and systemic responses to IMI challenge. Details on local and systemic responses to IMI challenge with *S. uberis *are described elsewhere [14]. Briefly, in response to IMI challenge, heart rate and body temperature were significantly elevated (*P *< 0.001), and there was a trend (*P *= 0.058) for increased respiration rate. All cows developed mastitis after IMI challenge with *S. uberis*. Clinical signs, such as flakes, watery, or yellow colored mammary secretions were observed after inoculation (i.e., between 24-36 h post-inoculation). Milk somatic cell count (**SCC**) from challenged quarters was increased (*P *< 0.001; 5.41 ± 0.17 log_10 _cells/mL) by 20 h when compared to 0 h post-inoculation (3.9 ± 0.17 log_10 _cells/mL). An overall increase (*P *< 0.001) in growth of *S. uberis *was observed in inoculated quarters. By 12 h post-inoculation, *S. uberis *was recovered from all challenged quarters and shedding continued through 36 h post-inoculation similar to results of others [15,16]. Details on individual quarter SCC and shedding of *S. uberis *are shown in Figure S1 in Additional File 1.

Based on previous work in our laboratory [17], as well as challenging 4 'test' cows prior to our experiment, peak clinical signs based on heart rate, respiration rate, milk secretion, shedding of *S. uberis *and, most importantly, increased SCC occurred between 24-36 h post-challenge. Therefore, biopsies were taken prior to peak clinical signs to be more confidient that the majority of gene expression data was attributed to MEC and not infiltrating neutrophils. Additionally, after biopsy (for details see Additional File 1), tissue (≥ 0.5 g) was blotted with sterile gauze to remove any visible milk secretions, and visible connective tissue was cut off and removed. The infiltration of immune cells was assessed via specific macrophage and neutrophil gene markers present on the bovine microarray (Figure S2 in Additional File 1). The data indicated an absence or a very slight increase in infiltration due to IMI by 20 h post-inoculation. Therefore, most of the responses in the present analysis must be attributed to MEC; however, resident macrophages constitute ca. 5% or more of the parenchyma tissue [18] and increased activity of those cells could be detectable via gene expression, particularly for genes with low inherent expression in MEC.

### Differential expression of genes in *S. uberis*-infected quarters

A total of 2,102 oligonucleotides (1,808 annotated genes) were differentially expressed (**DEG**) in response to IMI infection (False Discovery Rate; **FDR ***P *≤ 0.06; unadjusted *P *≤ 0.01) (see Additional File 2 for data and statistics). Of these, 1,082 genes were up-regulated and 1,020 genes were down-regulated. From Ingenuity Pathway Analysis^® ^(**IPA**; Ingenuity Systems, Inc.), a total of 1,675 genes were mapped or recognized based on annotation to a human or mouse ortholog within the IPA Knowledge base. Of these, 1,506 genes were eligible for generating networks and 1,264 genes were mapped to known functions and/or pathways based on published data across several species, including human, rat and mouse (see Additional Files 3, 4, 5, 6, 7, 8 and 9). When a 1.5-fold change cut-off was applied, among 173 oligos which passed this additional criterion, 158 genes were eligible for networks and 143 for functions and/or pathways analysis. Functions, pathways, and gene networks for the analysis of 1.5-fold change cut-off generated via IPA are presented in Additional Files 10, 11, 12, 13 and 14.

### qPCR

Table S1 in Additional File 1 lists genes selected for qPCR. A total of 58 genes were analyzed: 37 were differentially expressed with microarrays, 6 genes were not present on the microarray platform, and 15 genes were not significant at an FDR ≤ 0.06. The latter genes were selected based on their involvement in immune response or lipid metabolism. Among DEG, 78.3% (29 out of 37 genes) correspond to results of microarrays. Considering all the genes tested with qPCR we observed that *ACP2 *and *BAX *had responses opposite to microarray results, *ADFP, ADRB2, ALOX5AP, ANXA1, C3, C1QC, HMOX1, IL10, IL1B, INSR, NR3C1, PRKCB1, SAA3, SOCS2*, and *TNF*, which were not significantly affected in microarrays (FDR > 0.06) were found to be affected significantly by qPCR (*P *≤ 0.05). In addition, *BAX*, *IL15, LALBA, SDHD*, and *VLDLR *were significant with microarray but not with qPCR. Quantitative PCR is a more sensitive method of for gene expression analysis, thus, the qPCR data instead of the microarray data were used for IPA analysis in all cases as we have done in previous work [19]. Six genes (*BNBD5, CASP8, COX1, INSIG1, IRAK1*, and *TRAF6*) measured via qPCR were not present on the microarray platform but have been shown to be involved in immune response or metabolic pathways in mammary tissue [7,20]. Of these, all but *COX1 *were up-regulated by IMI.

### Individual DEG

Table 1 shows the top 10 genes up-regulated (10- to >1,000-fold) after IMI challenge with *S. uberis*. All genes play major roles in immune response during infection including cytokines (*IL8, IL6, IL10, IL1B*, and *TNF*) and *SAA3 *(an acute-phase protein), as well as PMN adhesion selectin-L (*SELL*) and *LYZ *(involved in anti-microbial defense). The top down-regulated genes (-1.68 to -2.3-fold) after IMI are shown in Table 2. The primary functions of these genes included lipid metabolism (*LPIN1, LPL, CD36*, and *BTN1A1*) and cellular transport of minerals, particularly Zn and Cu (*SLC30A4 *and *SLC31A1*).

**Table 1:** List of top 10 up-regulated genes in mammary tissue due to IMI with *Streptococcus uberis*^1^.

Gene Symbol	Gene Name	Primary Functions	Fold Change
*IL8*	interleukin-8	Chemotaxis; neutrophil activation; G-protein coupled receptor protein signaling pathway; angiogenesis.	1054*
*IL6*	interleukin-6 (interferon, beta)	Acute phase response; B- and T- cell activation; neutrophil activation and apoptosis.	430*
*IL1RN*	interleukin-1 receptor antagonist	Inhibits activity of IL-1, IL-1α, and IL-1β.	103.3*
*SAA3*	serum amyloid a3 (mammary)	Acute phase response; antimicrobial activites.	64.1*
*TNF*	tumor necrosis factor-alpha	Acute phase response; pro-inflammatory immune response; regulation of cytokine secretion; insulin signaling; glucose metabolism.	44.9*
*IL10*	interleukin-10	Anti-inflammatory immune response; inhibits pro-inflammatory cytokine secretion; induces IL-1RN and soluble TNF receptor expression; negative regulator of antigen presentation.	27.8*
*PLAUR*	plasminogen activator, urokinase receptor	Localizes and promotes plasmin formation; involved in cell-surface plasminogen activation and localized degradation of the extracellular matrix.	18.7*
*LYZ*	lysozyme	Anti-microbial defense agent via binding to bacterial cell wall peptidoglycan cleaving beta [1-4]glycosidic linkages.	16.8*
*IL1B*	interleukin-1 beta	Acute phase response; neutrophil chemotaxis; induces pro-inflammatory cytokine production.	13.9*
*SELL*	selectin-l	Adhesion of leukocyte to endothelial cells.	10.0*

^1^Asterisk denotes qPCR data.

**Table 2:** List of top 10 down-regulated genes in mammary tissue due to IMI with *Streptococcus uberis*^1^.

Gene Symbol	Gene Name	Primary Functions	Fold Change
*LPL*	Lipoprotein lipase	Lipoprotein hydrolysis to allow fatty acid uptake	-1.98*
*CD36*	CD36 molecule	Binds long chain fatty acids and may function in the transport and/or as a regulator of fatty acid transport.	-1.91*
*LPIN1*	Lipin 1	Triglyceride synthesis; PPAR co-activator	-2.30*
*TRAF3IP3*	TNF receptor-associated factor 3 interacting protein	Primary functions unknown; Play role in cell growth via modulation of JNK pathway; Proapoptosis	-2.25
*SLC30A4*	Solute carrier family 30, (zinc transporter), member 4	Transport zinc out of the cytoplasm.	-1.91
*SLC31A1*	Solute carrier family 31, (copper transporter), member 1	Copper ion transmembrane transporter.	-1.76
*KRT19*	Keratin 19	Involved in structural integrity of epithelial cells.	-1.73
*PEG3*	Paternally expressed 3	Nucleic acid binding; Transcription factor activity; Zinc ion binding; Metal ion binding.	-1.71
*IGF2BP2*	Insulin-like growth factor 2 mRNA binding protein 2	Functions by binding to the 5' UTR of the insulin-like growth factor 2 (IGF2) mRNA and regulating IGF2 translation.	-1.71
*BTN1A1*	Butyrophilin, subfamily 1, member A1	Butyrophilin is major protein associated with milk fat droplet.	-1.68

^1^Asterisk denotes qPCR data.

### Functional analysis using IPA and gene ontology (GO)

The use of IPA (Additional Files 3, 4, 5, 6, 7, 8, 9, 10, 11, 12, 13 and 14) and GO (see Additional Files 15 and 16) on the entire list of DEG (2,102) or those with a 1.5-fold cut-off revealed marked activation of genes associated with immune-related and inflammatory-related functions as well as an overall inhibition of lipid-related functions.

In particular, the IPA analysis (Additional Files 3, 4, 5, 6, 7, 8 and 9) with all 2,102 DEG uncovered induction of a wide number of functions:

- proliferation of smooth muscle cells, endothelial cells (both constituent of blood vessels), lymphocytes, and fibroblasts

- apoptosis of immune cells but a likely inhibition for epithelial cells

- large recruitment and infiltration of immune cells, particularly PMN, but also smooth muscle cells and bone marrow cells

- quantity of nitrite, as well as release of lipid with a likely increase in glucose transport

- the synthesis of nitric oxide was markedly induced, together with the production of peroxide and hydrolysis of GTP

- remodeling of tissue

- adhesion and activation of immune cells

- angiogenesis

- cell cycle activity, with a likely increase in the differentiation of lymphocytes but a decrease in differentiation of muscle cells

- morphological changes in cells, particularly for leukocytes and fibroblasts, with large reorganization of the cytoskeleton and formation of blebbings

- strong activation of inflammation, but when considering the entire transcriptional and animal (e.g. blood cortisol) responses, the direction of gene expression suggested and overall decrease of inflammation

- likely inhibition of triacylglycerol (**TAG**) synthesis but an overall induction of lipid synthesis, particularly prostaglandins.

Besides immune-related functions which included binding and activity of IL-1, IL-8, IL-10, TNF-α and chemokines, the GO molecular function analysis (Additional File 15) uncovered an increase in binding of genes encoding heat-shock proteins, NAD, and tetratricopeptide repeat (**TPR**) domain of a protein, the latter being important in the regulation of vasorelaxation [21]. In addition, analysis highlighted a decrease in genes encoding cofactor binding, particularly Mn and Se, a reduction of protein Ser/Thr phosphatase activity, reduction of oxidoreductase by NAD/NADP, an increase in long-chain fatty acid ligase activity but reduction of several processes related to acyl-carrier proteins.

The biological processes most enriched in GO (Additional File 15) indicated a marked effect on cell signaling, mostly related to apoptosis (e.g., caspase activation). The immune response, and associated metabolism (e.g., nitric oxide synthesis) and response to wounding, were the most affected and activated processes. Proliferation was highly increased in immune but also in endothelial cells. Protein metabolism also was highly activated, with protein transport being the most enriched process, particularly protein targeting which was increased. Interestingly, data indicated an increase in transport of proteins towards mitochondria. Data also suggested a marked increase in post-translational modification of proteins such as folding, methylation, and alkylation. The analysis indicated a sparing of the amino acid Ser, probably for protein synthesis, by inhibition of its catabolic utilization. Transcription was strongly activated, as well as post-transcriptional modification, while catabolism of DNA was inhibited. The transport of minerals, particularly di- and tri-valent cations (which include calcium and zinc), was largely increased. Fatty acid biosynthesis was evidently inhibited. Malate metabolism, both mitochondrial (for the TCA cycle) and cytosolic was highly-activated. There was also an inhibition of kinase activity. Lastly, as highlighted by IPA, the regulation of adhesion was highly activated.

The GO results for cellular components significantly affected during *S. uberis *infection (Additional File 15) revealed a strong morphological change of cells, mostly for the formation of filopodium, i.e. "microspikes" or cytoplasmic projections from migrating cells which play an important role in cell migration and wound healing [22]. Extrinsic component of membranes were increased in abundance with large effect on proteins present in the cytoplasm and organelles. Among those most enriched was the endoplasmic reticulum (**ER**), particularly the transport from nucleus to ER. Increased abundance also was evident for components of actin filaments, which agrees with the findings for cytoskeletal rearrangement reported above for GO and IPA. Interestingly, components of lysosomes and the Golgi were inhibited, as well as components of the vesicle membrane; whereas, ER-Golgi intermediate compartment components were induced. Components of the proteasome, involved in protein degradation, were also induced together with the MHC complex components. The increase in abundance of the phosphatase type 2A complex supports the increase in prostaglandin synthesis uncovered by IPA.

Overall, the analysis of DEG with ≥ 1.5-fold by IPA (Additional Files 10, 11, 12, 13 and 14) and GO (Additional File 16) provided results strikingly similar to the ones obtained using all DEG. This suggested that the functions found as significantly-enriched in the latter analysis included genes whose expression was highly affected. The GO analysis, however, allowed us to uncover a marked up-regulation in expression of genes for G-coupled receptors, cytokine and chemokine-mediated signaling, NFκB import into nucleus; whereas, down-regulated genes were associated with muscle development and organization. The significant enrichment of negative regulation of apoptosis and the inhibition of fatty acid metabolism via GO analysis also was noteworthy. GO analysis results for cellular components and molecular functions among DEG ≥ 1.5-fold (Additional File 16) confirmed the analysis that included all DEG.

### Canonical pathway analysis using IPA

**Figure 1:**
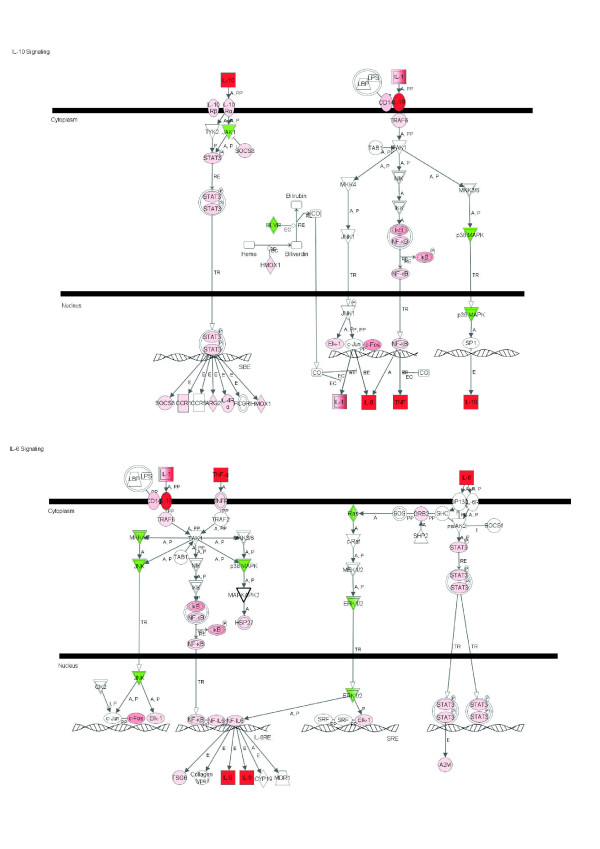
**IL-10 and IL-6 signaling pathways among 2,102 DEG due to IMI with *Streptococcus uberis***. Red denotes up-regulation and green down-regulation of the gene.

**Figure 2:**
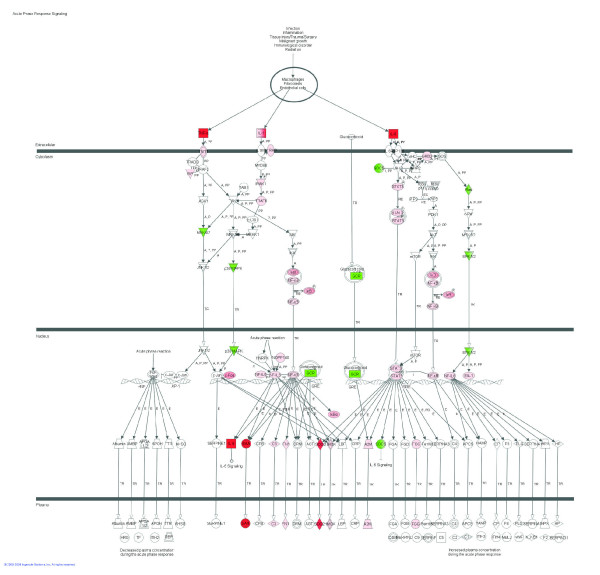
**Acute phase response signaling pathway among 2,102 DEG due to IMI with *Streptococcus uberis***. Red denotes up-regulation and green down-regulation of the gene.

**Figure 3:**
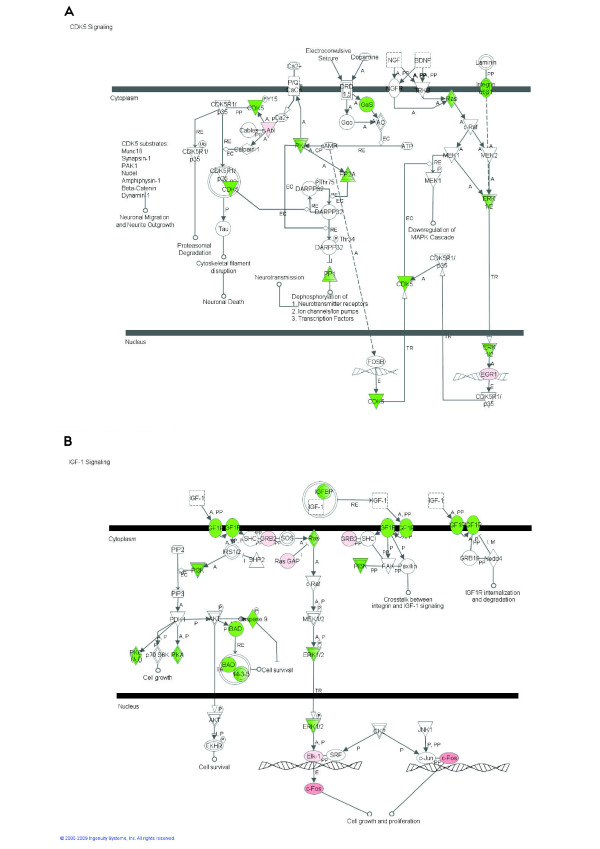
**Cyclin-dependent kinase (CDK) 5 (A) and Insulin-like growth factor (IGF)-1 signaling pathways (B) among 2,102 differentially expressed genes due to intramammary infection with *Streptococcus uberis***. Red/pink denotes up-regulation and green denotes down-regulation of the gene.

**Figure 4:**
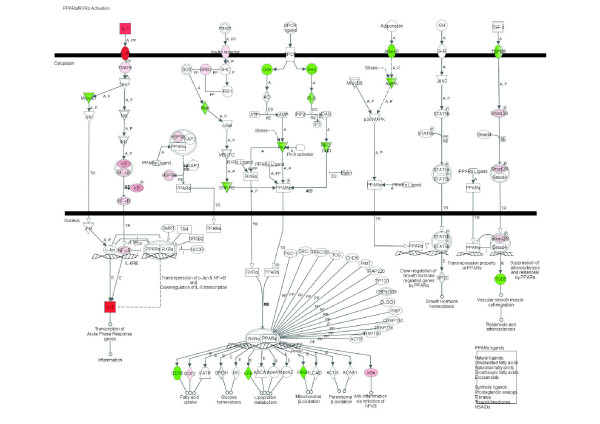
**PPARα/RXRα signaling pathway expression among 2,102 DEG due to IMI with *Streptococcus uberis***. Red denotes up-regulation and green down-regulation of the gene.

The top canonical signaling and metabolic pathways within all DEG (i.e., 2,102) are reported in Table 3. Detailed images of selected pathways are shown in Figures 1, 2, 3 and 4 and all pathways are shown in Additional Files 6, 7, 8, 9 and 12. As for the functional analysis (Additional Files 3, 4, 5, 10, and 11), most of the pathways affected via IPA analysis were related to immune or inflammatory functions. It was striking that signaling pathways were the most affected and very few, with lower significance, among all DEG were metabolic pathways.

**Table 3:** Top signaling and canonical pathways from Ingenuity Pathways Analysis (IPA) among the 2,102 DEG^1^.

Ingenuity Canonical Pathways	*P*-value	Ratio	Genes	↑/↓	Effect	Function (from IPA)
**Signaling pathways**						
ERK/MAPK Signaling	7E-09	0.26	50	33/17	**↓**	Induce growth and differentiation
IL-10 Signaling	8E-08	0.35	25	22/3	**⇑**	Limit and terminate the inflammatory
Glucocorticoid Receptor Signaling	1E-07	0.21	61	42/17	**↓**	Regulate immune, metabolic, cardiovascular and behavioral functions
IL-6 Signaling	2E-05	0.28	27	24/3	**⇑**	Regulator of acute-phase responses and a lymphocyte stimulatory factor
Ceramide Signaling	2E-05	0.29	26	16/8	**⇓**	Regulation of apoptosis and inflammation
Ephrin Receptor Signaling	3E-05	0.21	40	23/17	**⇓**	Axon guidance, cell migration, angiogenesis and synaptic plasticity
PI3K/AKT Signaling	3E-05	0.23	31	22/9	**⇓**	Pathways of cytokines, growth factors and other extracellular matrix proteins
PDGF Signaling	5E-05	0.28	22	15/7	**↑**	Growth, survival and function especially in connective tissue
Axonal Guidance Signaling	1E-04	0.17	67	39/28	**⇓**	Help navigate the axon to its final destination
Chemokine Signaling	2E-04	0.28	21	11/10	**⇓**	Act through cell surface receptors to induce inflammation and many processes
Acute Phase Response Signaling	2E-04	0.21	36	31/5	**⇑**	Inflammatory response
LXR/RXR Activation	2E-04	0.24	20	14/6	**⇓**	Mediate the biological effects of retinoids on lipid metabolism and inflammation
fMLP Signaling in Neutrophils	2E-04	0.21	27	17/10	**⇓**	Regulate many neutrophil functions such as migration and phagocytosis
Aryl Hydrocarbon Receptor Signaling	2E-04	0.21	34	16/16	**↓**	Xenobiotic metabolism, cycle progression, cell proliferation, and apoptosis
PPARa/RXRaActivation	2E-04	0.20	37	21/16	**⇓**	Fatty acid metabolism and anti-inflammatory
IL-3 Signaling	3E-04	0.27	20	11/9	**↑**	Regulates hematopoiesis
CDK5 Signaling	4E-04	0.25	23	15/8	**⇓⇓**	Post-mitotic processes such as neuronal activity, migration, and neurite outgrowth
Apoptosis Signaling	4E-04	0.25	25	14/9	**↔**	Apoptosis of programmed cell death
IGF-1 Signaling	4E-04	0.24	23	12/11	**⇓⇓**	Promotes cell proliferation, growth and survival
Recognition of Bacteria and Viruses	5E-04	0.24	21	17/4	**⇑**	Recognize conserved microbial structures or pathogen-associated molecular patterns
**Metabolic pathways**						
Nicotinate and Nicotinamide	2E-02	0.14	18	9/9	**⇓**	Synthesis and oxidation/reduction of NADH/NADPH
Pyruvate	2E-02	0.10	17	9/6	**↓**	
Arginine and Proline	4E-02	0.08	14	9/5	**↔**	

The *P*-value denotes the significance of the enrichment of a function within the DEG adjusted by Benjamini and Hochberg's FDR ≤ 0.06

^1^Shown also are the ratio (DEG/number of genes in the pathways), the total number of DEG in the pathway (Genes), the number of up- (**↑**) and down- (**↓**) regulated DEG in the pathway, the overall effect on the pathways (denoted by **⇑⇑ **= strongly activated; **⇑ **= evidently activated; **↑ **= likely activated; **↓ **= likely inhibited; **⇓ **= evidently inhibited; **↔ **equilibrium) see details in Additional Files 6, 7, 8, and 9.

Among signaling pathways, those data revealed a unique landscape where induction of certain pathways which limit the inflammatory response [e.g., induction of *IL-10 Signaling *(Figure 1), apparent inhibition of *Chemokine Signaling *and *fMLP Signaling in Neutrophils*] was coupled with activation of certain pathways which promote the inflammatory response (e.g., IL-6 and acute phase response signaling; Figures 1 and 2, respectively). Glucocorticoid signaling (Table 3 and Additional Files 7 and 8) and other pathways related to the latter, such as ERK/MAPK and PI3K/AKT signaling (Table 3 and Additional Files 7 and 8), were highly enriched and were likely induced except ERK/MAPK signaling, which the detailed analysis did not indicate induction or inhibition. It was evident that most of the pathways represented within the DEG were inhibited. Pathways strongly inhibited were *CDK5 Signaling *(Figure 3A), related to neuronal function, and *IGF1 Signaling *(Figure 3B) related to proliferation and survival of cells. There also were other pathways related to neuronal activity that were evidently inhibited such as *Ephrin Receptor Signaling *and *Axonal Guidance Signaling *(Additional Files 7 and 9). Pathway analysis also revealed a decrease of *Ceramide Signaling *for apoptosis, which suggested that the significant response related to apoptosis uncovered by the functional analysis, was not related to ceramides. Interestingly, results showed that two signaling pathways related to lipid metabolism (*LXR/RXR *and *PPARα/RXRα Signaling*; Figure 4; Additional Files 7, 9 and 12) were inhibited by IMI.

There were few significant metabolic pathways within the 2,102 DEG which we considered marginally significant (FDR ≤ 0.06; Table 3 and Additional File 6). Details of the pathways indicated a decrease in synthesis and oxidation/reduction of NADH and NADPH, an overall decrease of pyruvate metabolism, and an evident increase in utilization of pyruvate for the TCA cycle through formation of malate coupled with decrease of pyruvate utilization for other processes (e.g., lipid synthesis). Arginine and Pro metabolism also were affected. More detailed analysis of this pathway indicated a decreased utilization of Arg for catabolism and an increased utilization of this amino acid for protein synthesis. Similarly, there was evidence for increased utilization of Pro for other metabolic purposes besides synthesis of protein (Additional File 6).

The top canonical pathways among DEG affected by ≥ 1.5-fold are shown in Table 4 and Additional File 12. These included *IL-10 Signaling*, *LXR/RXR Signaling*, *IL-6 Signaling*, and *Glucocorticoid Receptor Signaling*. The majority of genes with ≥ 1.5-fold change within these pathways were up-regulated. All those pathways were related to immune response and lipid metabolism, with a general induction of immune-related pathways and inhibition of lipid-related ones. This apparently negative association between immune response and lipid metabolism is supported by the primary functions that were observed in IPA and GO when all DEG were considered (Additional Files 3, 4, 5 and 15). *Interleukin-10 Signaling *was the primary canonical pathway affected by IMI challenge with *S. uberis *among those genes with >1.5-fold change in expression (Figure 1 and Additional File 12). The triggering receptor expressed on myeloid cells 1 (*TREM1*) belongs to the Immunoglobulin (Ig) family of cell surface receptors and is selectively expressed on blood PMN, monocytes and macrophages. TREM-1 lacks known signaling motifs in the cytoplasmic domain and thus activation by TREM-1 is mediated by a transmembrane adaptor molecule DNAX-activating protein 12 (DAP12), leading to proinflammatory immune responses. Overall, IPA analysis indicated that early response factors during IMI with *S. uberis *encompass large effect on expression of genes associated not only with immune response (i.e., *IL-10 and IL-6 Signaling*; Figure 1; Table 4) but also endocrine signaling (*Glucocorticoid Receptor Signaling*; Additional File 12; Table 4) in mammary tissue.

**Table 4:** Top 5 enriched canonical pathways among DEG with ≥ 1.5-fold due to intramammary infection^1^.

			Genes	
			
Canonical Pathway	Up/Down	#Genes/Total^2^	Up-Regulated	Down-Regulated
*IL-10 Signaling*	19/0	19/71	BCL3*^3^, CCL2, FOS*, HSPA8, IL1B*, IL1R2*, IL1RN*, IL6*, IL8*, IL10*, NFKBIA*, STAT3*, TNF*	
*LXR/RXR Activation*	11/4	15/85	ARG2, CCL2, CD14*, IL1B*, IL1R2*, IL1RN*, IL6*, IL18*, LDLR*, TLR4*, TNF*	CD36*, FASN, LPL*, SCD*
*IL-6 Signaling*	13/0	13/94	BCL3*, CD14, FOS*, IL1B*, IL1R2*, IL1RN*, IL6*, IL8*, IL18*, NFKB1A*, STAT3*, TNF*, TNFIP6	
*Triggering receptor expressed on myeloid cells 1 (TREM1) signaling*	11/0	11/69	CCL2, IL1B*, IL6*, IL8*, IL10*, IL18*, STAT3*, TLR2*, TLR4*, TNF*, TREM1	
*Glucocorticoid Receptor Signaling*	17/0	17/275	ANXA1*, BCL3*, FOS*, HMOX1*, HSPA5, HSPA8, IL1B*, IL1R2*, IL1RN*, IL6*, IL8*, IL10*, SMAD3, STAT3*, TNF*, VCAM1	

^1^One rear quarter of all mid-lactation Holstein cows (n = 10) was inoculated with 5,000 cfu of *Streptococcus uberis *(strain O140J).

^2^#genes/total = number of differentially expressed genes from microarray and qPCR analysis (≥ 1.5-fold change; FDR ≤ 0.06; *P *< 0.01) out of total # of genes associated with the canonical pathway according to Ingenuity Pathway Analysis.

^3^genes with * symbol = qPCR expression results for verification of genes on microarray as well as genes selected for qPCR analysis not present on microarray.

### Gene networks among DEG with ≥ 1.5-fold during IMI challenge

**Figure 5:**
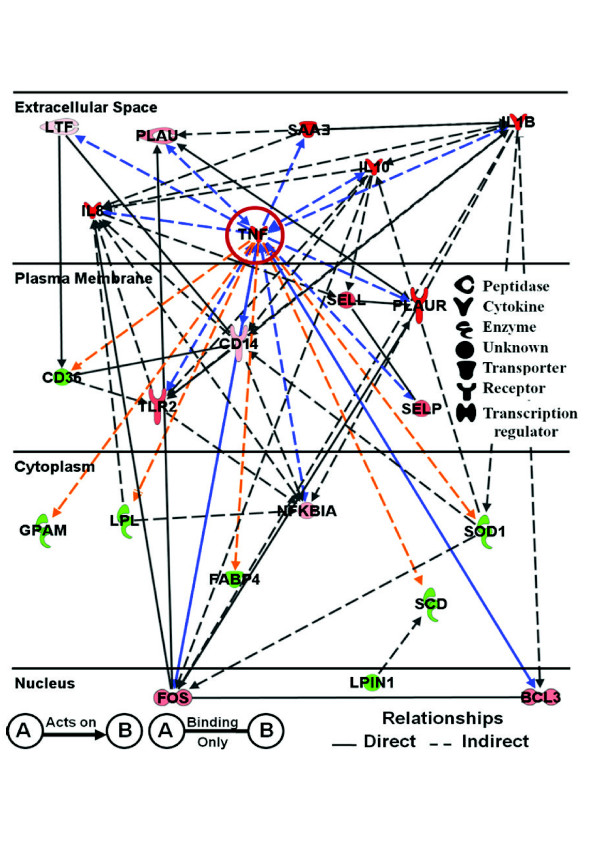
**Ingenuity Pathway Analysis^® ^network depicting relationships among genes involved in immune and metabolic responses due to IMI with *Streptococcus uberis***. Red denotes up-regulation and green down-regulation of the gene. Arrows highlighted in blue and orange represent the known positive and negative relationships with *TNF*, respectively.

We identified 19 networks (158 DEG) within IPA analyses that were associated with IMI challenge among the DEG affected by ≥ 1.5-fold (Additional File 13) based on microarray and qPCR. The top 5 networks were merged to evaluate relationships between individual DEG during IMI challenge with *S. uberis*. These networks included a total of 100 DEG involved in pathways and functions including *Immune Response, Immune Disease, Connective Tissue Disorders, Lipid and Carbohydrate Metabolism, Molecular Transport, Cell-To-Cell Signaling, Tissue Development, Cellular Development, and Immune and Lymphatic System Development and Function*. The results of merging those networks are shown in Additional File 14. Within this larger network, a subset of DEG (22) identified as having the greatest fold-change in expression that play major roles in either immune response and/or lipid and carbohydrate metabolism are shown in Figure 5. These genes encode cytokines (*TNF, IL8*, and *IL1B*), lipid metabolism-related genes (*CD36*, *FABP4*, *GPAM, LPIN1*, *LPL*, and *SCD*), acute phase proteins (**APP**; *SAA3*), transcription regulators (*BCL3*, *FOS *and *NFKBIA*), receptors (*CD14, TLR2*, and *PLAUR*), peptidases (*PLAU *and *LTF*), and others such as *SELP, SELL*, and *SOD1 *(see Table S1 for details about those genes). All genes in this network (Figure 5), except for *GPAM *and *FABP4*, were verified by qPCR (Table S1). Of these, only *SAA3 *had results opposite of microarrays. However, *SAA3 *was not significantly affected with microarray (FDR = 0.66; -1.03-fold change).

Of the 15 genes up-regulated (red) within the network, all play a role in some aspect of the immune response including cytokine activity (*IL10, TNF, IL8*, and *IL1B)*, cell adhesion (*SELL *and *SELP*), immune activation (*CD14 *and *TLR2*), acute phase reaction (*TNF, IL1B*, and *SAA3*), apoptosis (*BCL3*), and plasminogen metabolism (*PLAU *and *PLAUR*). Interestingly, induction of plasminogen metabolism has been used as an indication of virulence of *S. uberis *associated with bovine mastitis [23,24]. Of the 7 DEG down-regulated (green), the majority are involved in milk fat synthesis (e.g., *SCD, LPL, GPAM*, and *LPIN1*). The network revealed that *TNF*, at least judging by human/rodent data within the IPA knowledge base, has both direct and indirect positive relationships (blue arrows) with DEG involved in immune response and negative relationships (orange arrows) with DEG associated with lipid metabolism.

## Discussion

### Immune system response genes

#### General considerations

The microarray analysis clearly indicated that the mammary gland after a 20 h inoculation with *S. uberis *experienced a wide transcriptional response, which encompassed > 2,000 genes. Overall, the functional analysis uncovered that few functions were significantly affected, i.e. immune response was clearly the most affected and induced followed by cell proliferation/cycle/death and transport of protein and ions both of which were induced. In contrast, lipid metabolism was inhibited. Cell proliferation was seemingly or evidently induced in IPA analysis but GO analysis revealed that regulation of cell proliferation was not clearly towards induction, suggesting that the process of proliferation was probably increased for certain cells (e.g., immune and endothelial cells) but was not important for others. The overall picture from IPA and GO analyses captured the most affected functions but did not provide information of the potential mechanisms at play. The use of well-established pathways (i.e., canonical pathways) together with information about single genes provided additional means to unravel the mechanisms controlling the mammary response to IMI before peak clinical signs.

#### Top up-regulated DEG

Numerous cytokines involved in the immune response were significantly up-regulated in mammary tissue during IMI challenge with *S. uberis*. Genes coding for the cytokines *TNF, IL6*, and *IL1B *were among the top DEG (Table 1). Furthermore, among the list of all DEG (i.e., 2,102) there were several up-regulated DEG that belong to pro-inflammatory pathways including *CD14*, *TRAF6*, *NFKBIA*, *NFKB2*, and *STAT3 *(Figure 1). Functional analysis with GO and IPA clearly underscored the induction of inflammation as well as cytokine binding in mammary tissue from IMI (Figure 1 and Additional File 15). Other cytokines or cytokine receptors up-regulated during *S. uberis *IMI included *IL18 *and *IL1R2 *(verified via qPCR; Table S1). Interleukin-18 (IL-18) can induce interferon gamma (IFN-γ) production from T cells and, in combination with IL-12, IL-18 can inhibit IL-4-dependent immunoglobulin (Ig) E and IgG1 production as well as activate IgG2a secretion by B cells [25]. However, microarray analysis indicated that expression of *IL12 *and *IFNG *was not significantly altered during IMI challenge with *S. uberis *and that *IL4R *expression increased (1.26-fold; Additional File 2). This may indicate that the up-regulation of *IL18 *had minimal downstream affects on the innate immune response to *S. uberis*. Similar results were observed by Yang et al. [26] where *IL18 *expression, but not *IFNG*, was up-regulated in MEC after IMI challenge with *Staphylococcus aureus *(***Staph. aureus***).

The chemokine *IL8 *had the greatest change in expression resulting in a fold-change of 1,054 in infected vs. control quarters (Table S1). The importance of this protein and its related functions was underscored by GO molecular function analysis both when the entire DEG or those with >1.5-fold were analyzed (Additional Files 2 and 15). This chemokine is induced upon stimulation of TNF or IL-1 (Figure 1) and serves as a primary chemoattractant during the innate immune response, thus, playing a major role in the chemotaxis of PMN. Therefore, it seems logical that the dramatic increase in *IL8 *expression would occur before peak clinical signs of mastitis. Swanson et al. [8] did not observe a significant change in *IL8 *expression in bovine mammary tissue after IMI with *S. uberis *(Strain 233); but increased *IL8 *mRNA expression has been reported in primary isolates of bovine MEC after challenge with *Escherichia coli *(***E. coli***) [27]. With regards to results of Swanson et al. [8], mammary tissue was collected between 60-132 hours post-inoculation when peak clinical signs already had occurred. In our study, mammary biopsies were performed prior to peak clinical response and prior to the major influx of PMN into the mammary gland (supported by gene markers analysis, Figure S2 in Additional File 1), milk compositional changes and clinical signs of mastitis [14], and our previous work using this dose and strain of *S. uberis *[17].

#### The anti-inflammatory IL-10 and pro-inflammatory IL-6 pathways are activated before peak clinical signs

*Interleukin-10 Signaling *was among the primary canonical pathways affected by IMI challenge with *S. uberis *(Table 3 and 4, Figure 1, and Additional Files 7 and 12). The binding of the IL-1 cytokine family to the IL-1 receptor mediates the activity of *TRAF6 *(tumor necrosis receptor-associated factor 6; Figure 1 and Additional File 12), leading to activation of the p38 MAPK signaling pathway that ultimately leads to increased transcription of *IL10*. Despite a significant down-regulation of p38 MAPK (i.e. *MAPK12*; -1.22-fold; Figure 1) during IMI, the observed 13.9-fold up-regulation of *IL1B *and 38.9-fold up-regulation of *IL1R2 *probably overcame that response and also might have been sufficient to account for the fact that JAK1 expression was down-regulated (-1.17-fold; Figure 1). Interleukin-10 is an anti-inflammatory cytokine that blocks NF-κB activity, which leads to suppression of pro-inflammatory mediators such as *TNF, IL6*, and *IL1*. Expression of 22 out of 25 putative components (71 total in IPA) of the IL-10 signaling pathway present in our microarray platform were moderately but significantly up-regulated (Figure 1). Of interest was the mild up-regulation of *STAT3 *(ca. 3-fold; Table S1) which in turn is known to activate *SOCS3 *and activate IL-6-signaling [28]. Despite the marked up-regulation of *IL10*, our results of pathway analysis were indicative of more pronounced inflammation and probably hampered IL-10 anti-inflammatory activity.

*Interleukin-6 Signaling *was a major pathway affected by IMI challenge with *S. uberis *(Tables 3 and 4 and Figure 1). Several genes overlap between *IL-6 *and *IL-10 Signaling*, including an up-regulation of *TNF, IL1B, NFKBIA, STAT3, TRAF6*, and *FOS *and down-regulation of *MAPK12*. Expression of *IL6 *occurs via the NF-κB signaling pathway. Interleukin-6 is a pro-inflammatory cytokine that is also involved in acute-phase protein signaling (Figure 2). The coordinated up-regulation of genes involved in both *IL-6 *and *IL-10 Signaling *during IMI with *S. uberis *is suggestive of an ability of the immune system to generate a pro-inflammatory response via the *IL-6 Signaling Pathway *while attempting to control the severity and duration of the inflammation via the anti-inflammatory *IL-10 Signaling Pathway*. By far, however, our data suggested that the pro-inflammatory response via *IL6 *and *TNF *overrode the anti-inflammatory response via *IL10*.

IL-6 also has been shown to have anti-inflammatory capabilities through inhibition of IL-1β and TNF [29-31]. In our study, however, the signaling pathway through TNF and IL-1 appeared largely activated (Figure 1), with a more pronounced up-regulation of *NFKBIA *(in the pathways in Figure 1, IκB expression is determined by this gene, which is one of its components) than *NFKB2 *(this genes is a component of NF-κB), which suggested a potential inhibitory effect on the induction of survival genes via NFKB2 [32] and a control of inflammatory response. In the context of regulation of cell death/survival, it was evident that cell survival via enhanced growth and differentiation might have been inhibited due to IMI because the genes coding for phosphorylation enzymes in the ERK (extracellular-regulated kinase)/MAPK (mitogen activated protein kinase) signaling pathway, which is involved in control of a broad range of intracellular functions [33] were down-regulated (Table 3). These data suggested that signaling through phosphorylation (see also PI3K/AKT signaling; Table 3, Additional Files 7 and 12) was inhibited as a result of IMI. This latter finding was also observed by GO analysis (Additional File 15).

Our results regarding IL-6 and IL-10 support the work of Swanson et al. [8] who observed an up-regulation of the *IL6 *receptor (1.83-fold change) and *IL10 *receptor alpha (1.91-fold change) in bovine mammary tissue after *S. uberis *IMI. Similar to our data, Lutzow et al. [7] after IMI challenge with *Staph. aureus *observed an up-regulation (via microarrays) of genes involved in both IL-6 and IL-10 signaling including *IL1B, IL6, IL8, CD14, FOS*, and *NFKBIA*. In our study, we isolated whey from all infected quarters and analyzed samples for concentrations of IL-10, IL-1β, and TNF at 0, 12, and 20 h (time of biopsy) post-inoculation [14]. No significant changes in cytokine concentrations were observed by 20 h post-inoculation when compared to pre-infection levels (0 h). This may be attributed to the fact that the mammary biopsies were performed prior to peak clinical signs of mastitis in order to avoid tissue samples with elevated amounts of mRNA from infiltrating PMN. Unfortunately, the side effects of the biopsy procedure (e.g., blood clots) made it impossible to isolate whey from mammary secretions during peak clinical signs. However, Bannerman et al. [15] evaluated cytokine secretions in whey collected from mammary quarters challenged with *S. uberis *and observed elevated milk concentrations of IL-1β, IL-8, IL-10, IL-12, TNF, and IFN-γ compared with healthy quarters by ~30 h post-challenge.

#### Toll-like receptor signaling

Bacteria contain pathogen-associated molecular patterns (**PAMPs**) motifs, such as LPS or lipoteichoic acid (**LTA**), that are potent stimulators of innate immunity. Lipid A is considered the active motif for the PAMP activity of LPS from Gram-negative bacteria such as *E. coli *that stimulates the innate immune response and activates TLR-4 and the LPS-LPS binding protein-CD14 complex; however, the active motif for the PAMP activity of LTA (i.e. Gram-positive bacteria such as *S. uberis *and *Staph. aureus*) remains unknown. Regardless, TLR-2 protein is activated via LTA. The toll-like receptor (**TLR**) signaling pathway results in the synthesis of several pro-inflammatory cytokines (*TNF, IL1B*, and *IL6*) and chemokines (*IL8*). Although this pathway was not among the most significant in IPA analysis (Table 3 and Additional Files 6, 7, 8, 9 and 12), several genes involved in TLR signaling were up-regulated during IMI challenge including *TLR2, TLR4, CASP8, CD14, FOS, IRAK1, TRAF6*, and *NFKBIA*. All genes were verified via qPCR (Table S1). In addition, *TOLLIP *(toll interacting protein), a negative regulator of inflammation, was also significantly up-regulated (1.15-fold change; Additional File 2).

Several studies have evaluated gene expression profiles in mammary tissue or MEC lines after challenge with another Gram-positive bacterium, *Staph. aureus *[6,7]. Lutzow et al. [7] observed that *Staph. aureus *alters both TLR-2 and TLR-4 signaling pathways. They observed an up-regulation of *TLR2, FOS*, and *NFKBIA *during IMI challenge with *Staph. aureus *as well as *TLR4 *and *CD14*, both of which are primarily activated via LPS from Gram-negative bacteria such as *E. coli*. These researchers also observed an up-regulation of pro-inflammatory mediators including *TNF, IL1B, IL8*, and *IL6 *after IMI with *Staph. aureus*. However, Yang et al. [26] observed that IMI challenge with high doses of *Staph. aureus *(10,000 cfu; Strain 1027) failed to activate NF-κB signaling and the pro-inflammatory genes *TNF *and *CXCL8*. A "weak" immune response may be attributed to the virulence factors associated with this strain of *Staph. aureus*, because heat-inactivated *Staph. aureus *increased the expression of TLR signaling components and NF-κB activation [26]. The TLR-mediated NF-κB activation not only signals numerous pro-inflammatory genes but also other anti-microbial immune defense genes such as beta-defensins, which are oxygen-independent peptides that have potent anti-microbial activities [34]. Our data also showed a significant increase in expression of *BNBD5*, the most abundantly-expressed member of the beta-defensin family of bactericidal peptides in MEC (4.19-fold change; Table S1; Additional File 2) [6]. Our data support results from Swanson et al. [35], who found increased expression of lingual antimicrobial peptide (LAP), a member of the beta-defensin family, during IMI challenge with *S. uberis*.

Our microarray analysis demonstrated an increased expression of both *TLR2 *and *TLR4 *after IMI challenge with *S. uberis *compared with control quarters. However, Swanson et al. [8] observed an up-regulation of *TLR2 *but not *TLR4 *expression in bovine mammary tissue after *S. uberis *IMI. Increased expression of both *TLR2 *and *TLR4 *signaling pathways during IMI challenge with Gram-positive or Gram-negative bacteria has been observed in recent studies [7,26,36]. Most of these studies have examined TLR expression patterns in response to *E. coli *or *Staph. aureus*, both major pathogens associated with mastitis in the dairy industry. Goldammer et al. [6] reported an increased expression of both *TLR2 *and *TLR4 *(8-to-12-fold change) in bovine mammary quarters naturally infected with *S. aureus *when compared to healthy quarters. This response is supported by results of Yang et al. [26], where both *TLR2 *and *TLR4 *were up-regulated after IMI challenge with either *Staph. aureus *or *E. coli*. Similar results were also observed when bovine MEC were challenged with LPS [36], as well as in mammary tissue after IMI challenge with *Staph. aureus *(determined via microarrays) [7].

#### Other DEG involved with immune response

Other DEG of interest that were significantly up-regulated during IMI challenge with *S. uberis *included *HLA-DRA *(1.82-fold change; Table S1 in Additional File 1; Additional File 2) and *C1QC *(1.37-fold change; Table S1 in Additional File 1; Additional File 2). *HLA-DRA *codes for the major histocompatability complex type II (**MHC II**) DR alpha and is primarily expressed on T lymphocytes and macrophages. This gene is considered a candidate gene marker of disease resistance [37]. The role of MHC II in mammary tissue is unclear. Fitzpatrick et al. [38]observed expression of MHC II-positive cells in the connective tissue of the healthy mammary quarters and quarters infected with formalin-killed *S. uberis*; although individual cell identification was not conducted. Swanson et al. [8] reported an up-regulation of *HLA-DRA *(1.73-fold change) in bovine mammary tissue after *S. uberis *IMI. The MHC II complex presents antigen fragments to T-helper cells by binding to the CD4 receptor on T-helper cells. However, we did not detect differential expression of *CD4*. Although mammary tissue was thoroughly blotted with gauze to remove any visual milk secretions, it is possible that the expression of *HLA-DRA *may have been acquired through milk lymphocytes and macrophages present in mammary tissue during the biopsy. MHC II expression in MEC warrants further investigation. The observed up-regulation of *C1QC *was opposite to results from both Swanson et al. [8] who found down-regulation (-1.74-fold change) after *S. uberis *IMI and those of Günther et al. [27] who observed a 1.6 to 3.2-fold decrease in mRNA expression of factors associated with the C1 complex (e.g., *C1qA*, *C1qB, C1s *and *C1r*) in bovine MEC after challenge with *E. coli*. The complement component C1q is the first step in the initiation of the classical pathway of the complement cascade [39]. Researchers have not been able to quantify C1q concentrations in mastitic milk and primarily attribute this to its large size (900 kDa), which may render it impermeable to the mammary epithelium [40].

No current information is available on the use of the lectin pathway in the mammary gland during an IMI. Researchers have concluded that the mammary gland must lack the classical pathway and therefore must rely primarily on the alternative pathway of the complement system [39]. The initial step of the alternative pathway involves the cleavage of complement component 3 (**C3**) into fragments C3a and C3b. The expression of *C3 *(via microarray and qPCR) was significantly up-regulated (1.43-fold change) in mammary quarters infected with *S. uberis *and supports the work of Swanson et al. [8]. The C3 component has been quantified in mastitic milk [41]. C3 is also a downstream intermediate step involved with both the classical and alternative pathways of the complement system that ultimately leads to the assembly of the membrane attack complex (MAC complex), which consists of complement proteins C5a, C6, C7, C8, and C9. The membrane attack complex plays a role in the disruption of the bacteria cell walls during the immune response.

Two genes involved in inhibition of the complement cascade were significantly up-regulated in infected versus non-infected mammary quarters. These genes were *CD59 *(1.22-fold change) and *CD55 *(2.07-fold change) (Additional File 2). *CD59 *is involved in the inhibition of the assembly of the membrane attack complex. *CD55*, or the decay accelerating factor for complement, binds to both the C2-C4b complex of the classical pathway and the C3-Cfb complex of the alternative pathway. This binding accelerates their decay, disrupting the cleavage of C3 into C3b and C3a fragments, which leads to inhibition of the cascade and prevention of damage to host cells. To our knowledge, this is the first report of a significant up-regulation in expression of the *C1QC *gene from mammary quarters infected with *S. uberis*. Swanson et al. [8] observed an inverse relationship between *C1Q *expression (-1.74-fold change) and C3 (2.36-fold change) after *S. uberis *IMI. The researchers did not elaborate on the inverse relationship in gene expression patterns between *C3 *and *C1Q*. Further research related to the classical pathway of the complement cascade in the mammary gland is needed.

#### Cell proliferation, angiogenesis, and apoptosis

The overall functional analysis both in IPA and GO clearly indicated an induction of proliferation of several types of cells but in particular immune, endothelial, and muscle cells. In contrast, several significantly-enriched pathways related to proliferation/angiogenesis were strongly (e.g., IGF1 in Figure 3B and ephrin receptor in Table 3) or likely inhibited (Aryl Hydrocarbon Receptor signaling; Additional Files 7 and 9), with both the platelet-derived growth factor (**PDGF**) and PI3K/AKT signaling pathways likely induced (see below).

### Angiogenesis and inflammation: possible role of PDGF signaling and hypoxia

Platelet-derived growth factor (**PDGF**) refers to a family of dimeric isoforms that are important for growth, survival, and function especially in connective tissue [42]. Four different PDGF chains have been identified, the classical PDGF-A and PDGF-B and the more recent PDGF-C and PDGF-D isoforms. These isoforms that occur as homodimers or heterodimers (PDGF-AA, AB, BB, CC and DD) exert their effects by differential binding to two receptor tyrosine kinases [42]. Binding of PDGF induces dimerization and autophosphorylation of the tyrosine kinase receptors. Depending on the PDGF isoform involved, homo or heterodimers of the receptor are formed. It is interesting that paracrine PDGF-B signaling has a role in blood vessel formation (i.e., angiogenesis) and it is a potent effector of epithelial cancer growth [42]. The likely induction of PDGF pathway in our study might partly explain the induction of biological processes such as positive regulation of cell proliferation and vasculature development (Additional Files 15 and 16). In fact, signaling through PDGF might have counteracted the marked inhibition of the Ephrin receptor signaling pathway (Table 3), which also is a pro-angiogenic pathway [43].

A key factor contributing to angiogenesis and aberrant cellular growth (e.g., epithelial tumors) is hypoxia [43]. As cells outgrow their blood supply or are deprived of oxygen, a transcriptional response to hypoxia is initiated. Although several transcription factor pathways seem to be involved, most attention has focused on hypoxia-inducible factor 1 (*HIF1A*), which was up-regulated with IMI (Additional File 2). This is a heterodimer of two DNA binding proteins, HIF1A, and the aryl hydrocarbon nuclear translocator (*HIF1B*) [43]. In normoxia, HIF1A is unstable and rapidly degrades via the proteasome, but as oxygen tension drops below 2% (e.g., air is <20%), HIF1A is stabilized, translocates to the nucleus and interacts with HIF1B. The heterodimer initiate a complex transcriptional program via specific hypoxia response elements [43]. In our experiment, we observed up-regulation of many hypoxia-responsive genes as seen in non-ruminants [44], e.g. *HIF1A *and several others involved in glucose metabolism/glycolysis (e.g., *SLC2A3*, *GAPDH*, *LDHA*), growth factors/cytokines (e.g., *IL6*, *IL8*, *PDGFB*), oxygen transport and iron metabolism (e.g., *HMOX1*, *LTF*), as well as several other genes/transcription factors involved in wound healing and angiogenesis (e.g., *FOS*, *JUNB*) (Table S1; Additional File 2). It is also interesting that both pyruvate and lactate originating from anaerobic glycolysis in tissues are angiogenic [44]. In the context of our study, a greater uptake of glucose due to up-regulation of *SLC2A3 *coupled with a reduction in the need for TAG synthesis as well as lactose for secretion in milk (e.g., most lipogenic genes and *LALBA *were down-regulated) could have led to accumulation of pyruvate from glycolysis and might have played a role in promoting hypoxia. The accumulation of pyruvate seems to be supported also by the likely inhibition of pyruvate metabolism (Additional File 6).

Despite up-regulation of *HIF1A *and aryl hydrocarbon receptor (*AHR*) due to IMI, our pathway analysis revealed that the two signaling pathways associated with response to hypoxia (Ephrin receptor and Aryl hydrocarbon receptor) were for the most part inhibited (Table 3). In the case of the Aryl hydrocarbon receptor signaling pathway, it seems likely that the marked increases in *IL6 *and activation of *NFKBIA *(Additional File 2) were the main causes for overall inhibition of the pathway.

### The mystery of IGF1 signaling and inflammation

Insulin-like growth factor binding 1 (**IGF1**) is considered an anabolic hormone and plays a pivotal role in mammary development [45] and potentially in maintaining the epithelial cells during the declining phase of lactation [46]. However, there is no evidence of a role of IGF1 in lactating mammary tissue, at the least in bovine. In support of this, work from one of our laboratories has observed that IGF1 signaling is not among the significantly affected pathways in bovine mammary tissue during lactation and appeared to be inhibited (M. Bionaz, S. L. Rodriguez-Zas, R. E. Everts, H. A. Lewin, and J. J. Loor, University of Illinois, Urbana, unpublished results).

The IGF1 signaling pathway (Figure 3B; Additional File 7) was strongly inhibited after *S. uberis *challenge, suggesting "resistance" of mammary tissue to IGF1 during IMI. In the immune system, signaling via IGF1 is a crucial event resulting in postponement of apoptosis (increasing survival) of PMN through mediation of the PI3K signaling pathway [47]. If that holds true in mammary after IMI, our data suggest that apoptosis of PMN was probably substantial at 20 h post inoculation. The functional analysis in IPA indicated that apoptosis was significantly affected, with a balance between induction and inhibition (Additional File 15) but overall this process was likely induced particularly in macrophages (Figure S2). Furthermore, as indicated by GO analysis (Additional File 15), apoptosis occurred through caspase activation (Additional File 15).

To our knowledge a direct inhibitory effect of inflammation on IGF1 signaling has not been reported; however, modulation of IGF signaling by glucocorticoids in muscle was previously demonstrated [48]. This observation suggests a possible effect of corticoids prior to mammary tissue collection. However, plasma cortisol was not increased significantly in cows after IMI in the present experiment [14]. In summary, the inhibition of IGF1 signaling might have played a role in decreased immune cell survival, particularly macrophages. A possible inhibitory effect of glucocorticoids on this pathway cannot be excluded. The inhibition of IGF1 signaling after IMI in bovine mammary is a novel finding that still requires teleological explanation.

### Lipid metabolism and immune response

#### Integration of lipid metabolism and inflammation: possible role of LXR/RXR and PPAR signaling pathways

Both LXRs and PPARs are involved in the regulation of metabolic and inflammatory signaling [49,50]. *PPARA *is expressed in liver, brown adipose tissue, heart, and muscle tissue and plays a pivotal role in fatty acid catabolism [49]; whereas, PPAR-γ (*PPARG*) is highly expressed in adipose tissue and macrophages and primarily regulates adipogenesis [50,51]. PPAR-γ has been shown to be expressed in bovine mammary tissue and is also significantly increased during lactation [51]. *PPARA *and *PPARG *have anti-inflammatory properties [50,52]. *PPARG *has been shown to interfere with the transcription of pro-inflammatory factors such as STAT and NF-κB in macrophages [53].

In non-ruminant macrophages, studies have shown that ligand-activated LXR inhibits expression of genes involved with immune response [54]. Interestingly, studies have also shown that *TLR4 *activation in macrophages inhibits LXR signaling [55]. Activation of inflammatory signaling pathways and release of inflammatory mediators are fundamental to the diverse immune functions of macrophages, and the mammary gland possesses resident macrophages [56]. In addition to inducing genes involved in reverse cholesterol transport, LXR reciprocally represses a set of inflammatory genes after bacterial lipopolysacharide (**LPS**), TNF, or IL-1β stimulation [57]. Examples of such genes include those involved in generation of bioactive molecules such as NOS2A, IL-6, TNF, and IL-1β, the chemokines CCL2, and matrix metallopeptidases. We found that IMI resulted in marked up-regulation of *IL6 *(430-fold), *TNF *(45-fold), *IL1B *(14-fold), and *CCL2 *(3.3-fold) and moderate but significant up-regulation of *NOS2A *(1.2-fold) and *MMP7 *(1.4-fold; Table S1 and Additional File 2). As previously stated, most of the responses in the present study are likely attributed to MEC and potentially resident macrophages, which constitute ca. 5% or more of the parenchyma tissue [18]. It is possible that increased *NOS2A *expression may be attributed to resident macrophages. However, studies have reported increased expression of the endothelial (*eNOS*) and inducible (*iNOS*) forms of nitric oxide synthase in human [58] and murine [59] breast cancer tissue. The increased *TLR4 *expression after IMI in our study may partly explain the down-regulation of the genes involved with LXR/RXR signaling. The *TLR4 *response might have been driven via up-regulation of *IRF6 *(Additional File 2) [57].

Studies investigating the LXR/RXR signaling pathway in the mammary gland are sparse and have primarily focused on expression of genes involved in this pathway during murine lactation regardless of bacteriological status [60]. Mouse mammary microarray data [60] has suggested the potential involvement of two systems in controlling fatty acid metabolism. These include the LXR/RXR pathway controlling 1) β-oxidation of fatty acids via LXR (also known as *NR1H2*)/PPAR dimers; and 2) fatty acid synthesis involving the LXR/RXR dimer, which induce expression of the sterol regulatory element-binding proteins 1 (*SREBF1*) and 2 (*SREBF2*).

The lactating bovine mammary gland does not seem to oxidize long-chain fatty acids as a source of energy [61], thus, any involvement of LXR in bovine mammary tissue might be at the level of fatty acid synthesis and/or inflammation (as in non-ruminant macrophages) [57]. However, the expression of LXR in bovine mammary tissue only increased slightly during lactation relative to pregnancy and it was not among DEG (M. Bionaz, S. L. Rodriguez-Zas, R. E. Everts, H. A. Lewin, and J. J. Loor, University of Illinois, Urbana, unpublished results). Those responses coupled with the lack of change in LXR expression due to IMI were suggestive of a minor role for LXR in mediating anti-inflammatory or lipogenic mechanisms in bovine mammary tissue.

Expression of *PPARA *is barely detectable in bovine mammary tissue (M. Bionaz, S. L. Rodriguez-Zas, R. E. Everts, H. A. Lewin, and J. J. Loor, unpublished results) and tends to decrease during lactation, which points to a minor role of this nuclear receptor in bovine mammary lipid metabolism. We recently showed that mRNA expression of *PPARG *was consistently up-regulated during lactation, suggesting that it could play a role in milk fat synthesis [51]. A role of *PPARG *in regulating bovine milk fat synthesis machinery was supported by recent results we obtained where treatment of MacT cells (bovine mammary epithelial immortalized cells) with rosiglitazone, a specific PPARγ agonist, resulted in coordinated up-regulation of genes involved in FA import (e.g., *CD36*), de novo FA synthesis (e.g., *ACACA, FASN, SREBF1*), and TAG synthesis (e.g., *LPIN1, SCD*) [62]. More importantly in the context of the present study, a recent study with PPARγ-knockout mice indicated that its absence increased utilization of long-chain fatty acids for synthesis of inflammatory lipids due to reduced TAG synthesis [63]. *PPARG*-knockout mice had a sustained increase in 12-lipoxygenase (i.e., *ALOX5AP*) activity from parturition through the end of lactation. Although we did not observe a significant effect of IMI on *PPARG *expression, up-regulation of *ALOX5AP *(ca. 6-fold; Table S1) might have been associated with increased synthesis of eicosanoids which are classical effectors of an inflammatory response. In addition, activation of PPARγ by specific agonists reduced synthesis of inflammatory cytokines in mammary epithelial cells, suggesting this nuclear receptor has an anti-inflammatory role in mammary tissue [64]. A 39-fold increase of *ALOX5AP *in mammary quarters challenged with *E. coli *in a recent study provides further support to the inflammatory role of *ALOX5AP *during an IMI [27].

Taken together, the above observations coupled with the down-regulation of PPARγ target genes point to PPARγ as a major player. The expression of this nuclear receptor appeared not to be affected by IMI (at the least from microarray data) but its activity probably was decreased as suggested by down-regulation of its known target genes. Similar to PPARα (Figure 4), the increase in NFκB activity might have inhibited PPARγ activity. Interestingly, insulin-induced gene 1 (*INSIG1*), which is involved in the inhibition of SREBP cleavage (i.e., inactivation of SREBP), and appears to be a PPARγ target gene in bovine mammary epithelial cells [62], was significantly up-regulated (1.5-fold change; Table S1). These data suggested that *INSIG1 *is not only under control of PPARγ but likely contributed to reduced milk fat synthesis through blockage of SREBP1 cleavage, i.e. both *SREBF1 *and *SREBF2 *are moderately up-regulated during lactation in bovine mammary tissue and could be involved in lipid synthesis through activation of acetyl-coenzyme A carboxylase alpha (*ACACA*) and fatty acid synthase (*FASN*) [51]. Unfortunately, the IPA Knowledge Base does not contain specific PPARγ pathways, thus precluding a definitive conclusion about the pivotal role of PPARγ. It is important to note that a possible role of PPARα cannot be excluded because specific PPARα co-activators or up-stream factors were down-regulated (Figure 4).

An enzyme linked to the LXR/RXR and PPARG pathways via SREBP1 in non-ruminant liver and adipose is stearoyl-CoA desaturase (*SCD*), which plays an essential role in TAG synthesis by catalyzing the synthesis of oleic acid via desaturation of stearic acid [65]. Oleic acid serves as a primary substrate for fatty acid binding protein 4 (*FABP4*) [66], and previous work in our laboratories proposed that *FABP3 *provides stearic acid, and other substrates, to *SCD*, which then provides oleic acid for *FABP4 *[51]. Expression of both *FABP3 *and *FABP4 *was down-regulated in infected versus control mammary quarters (-1.46 and -1.55-fold change, respectively). Expression of *SCD *was also inhibited in *S. uberis*-infected quarters (-1.64-fold change). Impaired PPARγ signaling might have been associated with the down-regulation of these lipogenic enzymes, either through down-regulation of *SREBF1 *or directly through decreased binding to response elements (e.g., *SCD *and *FABP4*).

Our findings highlighted a potential relationship between PPAR and LXR, two master regulators of lipid metabolism and inflammatory responses in non-ruminants [57]. The relationships between those two nuclear receptors with inflammatory conditions appear to be in two directions, i.e. their expression/activity is decreased by inflammation in mouse liver [67] and kidney [68], and an increase in their activity/expression leads to an anti-inflammatory effect [57]. Overall, our results indicated that IMI with *S. uberis *inhibited activity of LXR/RXR and PPAR signaling during IMI, suggesting that the anti-inflammatory effect of those pathways was not at play. We suggest that PPARγ signaling plays a primary role in mammary tissue but the activity of this nuclear receptor was probably reduced. The overall repression of lipogenic genes in *S. uberis *infected mammary quarters and the mechanisms involved in LXR/RXR or PPAR signaling and the fatty acid switch in the mammary gland during IMI challenge have not been elucidated and require further investigation. PPARγ has a pivotal role in immune cells as well, increasing their ability to face infections [69]. A possible role of PPARγ activation in reducing inflammation in mammary gland tissue has been previously suggested based on in vitro data [64] and our results support such a view.

#### Ceramides, inflammation, and lipid metabolism

Ceramide, which is involved in cell signaling, cell cycle, and regulation of protein transport from ER to Golgi, is one of the most studied sphingolipids in nature [70]. Other sphingolipids with signaling roles include sphingosine (**Sph**) and sphingosine-1-phosphate (**S1P**), which can activate NFKBIA and a cascade of inflammatory genes (Figure 1; Additional File 2) [71]. Although minor compared with TAG, sphingolipids are the third most important lipid component in bovine milk fat [72]. Formation of the milk fat globule membrane relies on sphingolipid and cholesterol availability, thus, coordinated synthesis of both compounds is pivotal to milk lipid droplet formation/secretion. Mammary tissue synthesizes sphingolipids de novo [72] from palmitoyl-CoA, leading to ceramide formation and incorporation into sphingomyelin. Thus, palmitic acid used for ceramide synthesis in mammary appears a required step and also might represent a regulatory point for FA synthesis because ceramides can inhibit this process by blocking the activity of AKT/PKB [73].

Our data revealed that ceramide signaling was markedly down-regulated (Table 3) potentially through the action of *TNF *(Table S1, Additional File 2). Based on the observed downregulation of lipogenic genes (e.g., *ACACA, FASN*; Table S1, Additional File 2) as well as serine palmitoyl transferase (*SPTLC2*; Additional File 2) it was apparent that ceramide synthesis was decreased, which likely explains the down-regulation of other genes that are part of its signaling pathway (Additional Files 7 and 9). The details of the pathway indicated a reduction of ceramide synthesis from sphingomyelin through activity of neutral sphingomyelinases sphingomyelin phosphodiesterase. In addition, the decrease in expression of genes involved in long-chain fatty acids import (e.g., *CD36, LPL*) and de novo fatty acid synthesis (e.g., *ACACA *and *FASN*) had probably reduce the amount of available palmitate for synthesis of ceramide. From our combined results, production of ceramide did not seem to be induced by pro-inflammatory state during IMI, but probably decrease. In addition, we observed an overall inhibition/decrease of ceramide downstream signaling, which clearly indicated that during IMI ceramide is not involved in apoptosis.

#### Significance of the immune response and milk fat synthesis

The negative relationship between DEG involved with immune response and milk fat synthesis may serve several beneficial purposes for the immune system within the mammary gland. First, the ability of phagocytes such as PMN and macrophages to engulf invading microorganisms is lower in milk when compared to PMN and macrophages that originate from the bloodstream. Milk phagocytes engulf milk fat globules instead of invading pathogens, resulting in a loss of pseudopodia needed for phagocytic capability [56]. Therefore, the less milk fat synthesized during an IMI the more likely that milk phagocytes will engulf invading bacteria instead of milk fat globules. As previously stated, *S. uberis *strain O140J has been shown to be more resistant to PMN phagocytosis and more capable of establishing infection when compared to a noncapsular strain [12,13]. Decreased expression of genes involved in *Lipid Metabolism *(using IPA Knowledge database) has also been recently reported after IMI challenge with *E. coli *[27]; and suggests that reduced lipid synthesis in the mammary gland may not be pathogen specific. In addition, microarray and qPCR analyses revealed a down-regulation of *LALBA *(-1.46-fold; Table S1), the rate limiting enzyme in lactose synthesis, which confirmed previous findings [74]. This may indicate that, at the time of biopsy (20 h post-inoculation), lactose synthesis was reduced as suggested by previously-reported milk whey analysis of mastitic cows [75]. A decrease in lactose synthesis might help the immune system by reducing substrate (i.e. lactose) for bacteria and also preventing a potential inhibition of PMN phagocytosis by lactose [76]. Inflammation reduces protein synthesis in muscle [77], but our transcript profiling did not indicate alterations in protein synthesis in infected compared with non-infected contralateral mammary quarters. However, there was an increase in expression of *CSN3 *(Table S1). Furthermore, the GO analysis uncovered an evident induction of transcription, post-translational modification, transport, and localization of proteins (Additional File 15). Those findings seemed to indicate that protein synthesis in milk should not have been decreased, but the large increase in transcription and protein metabolism was probably more related to increase synthesis and secretion of inflammatory-related proteins such as cytokines or acute-phase proteins. Unfortunately, quarter milk composition was not analyzed during the infection period; therefore, changes in milk fat, protein, and lactose could not be evaluated.

### Milk fat synthesis down-regulated DEG

The majority of DEG down-regulated by >1.5-fold (via qPCR or microarrays) in mammary quarters after IMI challenge with *S. uberis *were associated with lipid metabolism including lipoprotein lipase (*LPL*), *CD36*, lipin 1 (*LPIN1*), and butyrophilin (*BTN1A1*) (Table 2). However, the changes in gene expression were not as marked as those observed for up-regulated DEG. Swanson et al. [8] also reported down-regulation of genes involved with lipid metabolism (e.g., *LPIN1, APOB*, and *APOA2*) in bovine mammary tissue after IMI with *S. uberis*. This is further supported by Günther et al. [27], who observed a decrease in mRNA expression of factors associated with *Lipid Metabolism *(using IPA Knowledge database) such as *LPL*, *FASN *and *BTN1A1 *after IMI challenge with *E. coli*. Exogenous sources of non-esterified fatty acids (**NEFA**) and TAG in the circulation that are used for milk fat synthesis in the mammary gland originate from 1) chylomicra from dietary sources that enter the lymphatics and bypass the liver; 2) very low-density lipoproteins (**VLDL**) that are exported from the liver; or 3) NEFA bound to albumin that originate from adipose tissue [60]. The VLDL and chylomicra attach to the mammary endothelium by the enzyme LPL, which then hydrolyzes TG to fatty acids. Our results indicated that *LPL *was the third most down-regulated gene with a -1.98-fold change in expression versus control quarters (Table 2). This enzyme is located functionally in the capillaries, but is synthesized in parenchymal cells. Recent work in our laboratories has shown that *LPL *highest fold change in expression occurs during peak lactation (~60 days in milk) when compared to prepartum expression values [51]. Evidence also suggests a role for the VLDL receptor (*VLDLR*) in LPL activity [78], TAG metabolism and storage in adipocytes [79], and positive relationships with *LPL *expression during early lactation [51].

Another highly DEG during infection was *CD36 *(-1.91-fold change). This gene is highly expressed during early lactation in mammary tissue [51] and plays a role in fatty acid transport (i.e., translocation) across the plasma membrane of MEC, thus providing fatty acid for milk fat synthesis. Genes involved in TAG synthesis in the mammary gland, *LPIN1 *and *GPAM *(glycerol-3-phosphate acyltransferase, mitochondrial; -1.57-fold change; Additional File 2) were significantly down-regulated. *LPIN1 *had the greatest fold change in expression (-2.30-fold change) out of all DEG down-regulated in mammary from *S. uberis*-infected quarters.

The transport and export of newly-synthesized milk fat droplets is accomplished via BTN1A1, xanthine dehydrogenase (*XDH*), and adipophilin (*ADFP*) [80,81]. During early lactation, positive associations between *BTN1A1*, *XDH*, and *ADFP *were observed in healthy bovine mammary tissue [51]. Interestingly, these genes were found to have contrasting expression patterns during IMI with *S. uberis*. Within infected quarters, *BTN1A1 *was one of the top down-regulated genes (Table 2; -1.68-fold change) and *XDH *had modest down-regulation (-1.17; Additional File 2) when compared to control quarters. Furthermore, XDH can be converted to xanthine oxidase (**XO**) by reversible sulfhydryl oxidation or by irreversible proteolytic modification [82]. Production of XO is important for bactericidal activity against major bovine mammary gland pathogens including *E. coli *and *Staph. aureus *[82]. The specific response of bacteria species to XO and the resulting bacteria-dependent nitrosative stress demonstrated that, besides its central role in lipid droplet secretion, XDH plays a role in the mammary gland immune system [82]. The up-regulation of *ADFP *might have been a compensatory mechanism to sustain milk lipid droplet secretion. Further investigation is required to determine the specific role of lipid droplet proteins during IMI.

### Glucocorticoid signaling and related pathways

Glucocorticoids, a class of steroid hormones, exert dramatic effects on metabolism and immune response during periods of stress and lead to catabolism of lipids, carbohydrates, and proteins while increasing glucose availability in the bloodstream [83,84]. Glucocorticoids bind to the glucocorticoid receptor α (*NR3C1*) activating it. The activated glucocorticoid receptor inhibits inflammation through transcriptional repression of proinflammatory genes [85] and activates genes involved in the anti-inflammatory response (e.g. annexin I/lipocortin; *ANXA1*) and apoptosis (e.g. *BAX*) [86]. Despite lower mRNA for *NR3C1 *during infection (-1.33-fold; Table S1; Additional File 7), we observed up-regulation of *ANXA1 *(1.38-fold change; Table S1, Additional File 7). There was also a tendency towards an increase in expression of *BAX *(1.52-fold change; qPCR *P *= 0.06; Table S1).

The glucocorticoid signaling through glucocorticoid receptor is related to ERK/MAPK and PI3K/AKT [85]. Even though those pathways were overall likely induced by 20 h of IMI, the genes (mostly kinases) which are related those pathways were down-regulated. ERK/MAPK signaling seems to be essential for the anti-inflammatory effect of glucocorticoids via repression of p38 MAPK upon glucocorticoid treatment in mice [87]. Evidence of a modulatory effect of glucocorticoids on ERK/MAPK signaling pathways have been reported for human cancer cells [88]. Interestingly, in our case we observed a down-regulation of most of the genes coding for kinases (Additional File 2). Explanations for the down-regulation of kinases in the ERK/MAPK (Additional File 2) are not readily available. The PI3K/AKT is essential in the activation of NFκB by TNF [89], thus playing an inflammatory role in the tissue. In our case the details of the pathway indicate that the PI3K/AKT signaling was in favor of NFκB mediated transcription probably though induction by growth factors, while other down-stream effects were mostly inhibited (Additional Files 7 and 12). Based on the evident induction of proliferation and apoptosis, suggested by functional analysis (see above and Additional Files 3, 4, 5, 10, and 11) and details of the PI3K/AKT pathway, we can conclude that the likely induction of PI3K/AKT pathway is probably related to proliferation, apoptosis, and inflammation. The down-regulation of several genes in common with the glucocorticoid pathway seems to indicate that the relationship among those pathways is not playing a role in modulating inflammation at 20 h post-inoculation.

### Leukocyte extravasation and pain are transcriptionally regulated after IMI

The nervous system was not highly affected when considering functional analyses in IPA (Additional Files 3, 4, 5, 10, and 11) or GO (Additional Files 15 and 16); however, several pathways involved in neuronal outgrowth appeared significantly inhibited including Ephrin receptor, axonal guidance, and CDK5 signaling (Figure 3A). Most of those pathways are not strictly related to neurons.

Ephrin receptor signaling plays a role in attraction/repulsion, adhesion/de-adhesion implicated in axon guidance and migration of other cells beside neurons (e.g., leukocytes) but also plays a role in angiogenesis and synaptic plasticity. It has been suggested, based on multiple lines of evidence, that ephrin receptors play a direct role on inflammatory response [90]. The evidence points to a dual-phase pattern. In the early phase of inflammatory response the ephrin pathway is activated, inducing a decrease in adhesion between endothelial cells and epithelial cells. In a subsequent phase of inflammatory response the pathway is inhibited, increasing adhesion of circulating leukocytes to vascular endothelium and to epithelia of internal organs. The combination of the two allows the extravasation of leukocytes. Based on these effects, the evident inhibition of ephrin receptor signaling in our data (Additional Files 7 and 9) seems to indicate that at 20 h the inflammatory response was on its second phase, i.e. namely the adhesion and extravasation of leukocytes. Similar conclusions could be drawn from the axonal guidance signaling, which shares many of the Ephrin receptor pathway molecular networks (Additional Files 7 and 9). The importance of movement and invasion of leukocytes and cell regulation of cell adhesion was underscored also by functional analysis (Figure S2; Additional Files 15).

The CDK5 signaling is strictly related to neuronal physiology [91] and seems to participate in dendrite and synapse development [91], but also in nociception [92]. The nociceptive role of CDK5 signaling has been clearly demonstrated during induction of peripheral inflammation in mouse. During an inflammatory status, or other sort of pain, the level of calpains increases rendering the CDK5 more stable, a process which seems to increase the perception of pain [84]. Based on those previous results, the strong inhibition of CDK5 signaling at 20 h of IMI (Figure 3A) seems to indicate a degree of modulation or control of pain through decrease nociception within mammary tissue prior peak clinical signs of infection.

### Gene networks during IMI challenge with *S. uberis*

Figure 5 shows results from merging of the top 5 gene networks generated via IPA (i.e., networks most likely to have affected the system) describing relationships among DEG with ≥ 1.5-fold expression due to IMI. The merged networks encompassed genes involved in the immune response and lipid metabolism, with a central role of *TNF*.

#### Genes positively-associated with TNF

Not surprisingly, *TNF *was positively associated with pro-inflammatory mediators such as *IL8, IL1B*, and *NFKBIA*. The positive association with the anti-inflammatory cytokine *IL10 *further supports the co-regulatory mechanisms responsible for controlling the severity of the inflammatory response during an IMI [93-95].

The network in Figure 5 also shows a positive effect of *TNF *on the acute-phase protein *SAA3*. This supports the protein-level response observed in milk secretions from cows during IMI challenge with *S. uberis*, where milk SAA concentrations were elevated at 20 h post-inoculation when compared to pre-inoculation concentrations [14]. Serum amyloid proteins have immunological properties and the *SAA3 *isoform (i.e. *M-SAA3*) has been shown to be highly expressed in bovine MEC during mastitis [96]. Expression of mRNA for *SAA3 *in MEC is significantly enhanced in quarters challenged with LPS from *E. coli *or with *Staph. aureus *when compared to healthy quarters, indicating that the main source of SAA in milk during infection may be from MEC and not hepatocytes [4]. This premise is further supported by results of Eckersall et al. [97], who demonstrated that expression of *M-SAA3 *mRNA and haptoglobin (*HP) *mRNA were up-regulated during an experimental challenge with *Staph. aureus *and that mRNA for *M-SAA3 *was greater than that for *HP*. This increased expression of *SAA3 *and *HP *is specific to infected quarters because several studies have indicated that expression is minimal or not detectable in MEC from healthy quarters [98,99]. It is challenging to be able to distinguish between 2 gene isoforms with a 70-bp oligonucleotide on a microarray platform. The latest annotation of our microarray identified this oligo as both *SAA1 *and *SAA3 *and it clearly depends on the tissue type (i.e. liver or mammary) as to which isoform is primarily expressed. Upon verification, we confirmed that the sequencing results of primers were specific for *SAA3 *(Tables S3 and S4 in Additional File 1). IPA network analysis indicated that TNF-α protein has been shown to increase *SAA3 *mRNA expression in mouse granulosa cells [100] and in 3T3-L1 adipocyte cell lines [101]. SAA is primarily involved in the acute phase response and has been shown to increase leukocyte adhesion [102], but no relationships between *SAA3 *and genes encoding *SELL *and selectin-P (*SELP*) have been identified (Figure 5). However, *TNF *has been shown to affect both the expression and protein release of *SELP*, but not *SELL*, in murine endothelial cells (Additional File 15) [103].

Gene network analysis also shows that *TNF *has a positive relationship with *PLAU *and *PLAUR*. The enzyme PLAU is required for the normal repair of wounds originating on skin [104] and, as stated earlier, *S. uberis *can activate the conversion of *PLAU *to plasmin [23]. Plasmin increases during mastitis and hydrolyzes α_s_-casein, β-casein, and β-casein [105]. An increase in expression of κ -casein (*CSN3*; 1.82-fold change) was observed in *S. uberis *infected quarters (Table S1). Concentrations of κ-casein and plasmin were not quantified in milk secretions from infected quarters for this study, thus further research will be needed to investigate their correlations between mRNA expression and protein concentrations in milk as well as their specificity to *S. uberis*-associated mastitis.

In the nucleus, both *FOS *and *BCL3 *expression are stimulated by *TNF *(Figure 5). Expression of *FOS *was enhanced in human omental microvascular endothelial cells when incubated with TNF-α for 10 min [106], whereas *BCL3*, a nuclear protein primarily found in B lymphocytes, increased when human hepatocellular carcinoma cell lines (HepG2) were stimulated with TNF-α [107]. Another positive association within the network involved *LTF*, which competes for iron with invading microorganisms that require it for growth [108]. Watanabe et al. [109] observed a significant increase in LTF 4 h after intramammary infusion of recombinant bovine TNF-α.

Increased *CD14 *and *TLR2 *expression was observed in *S. uberis*-infected quarters when compared to healthy (control) quarters (Table S1). Hermoso et al. [110] observed an increase in *TLR2 *mRNA expression in carcinomic human alveolar basal epithelial cells (A549 cells) after stimulation with recombinant human TNF-α. Regarding *CD14*, TNF-α protein increased CD14 expression in rat Kupffer cells [111]. The CD14 molecule is primarily activated via the PAMP sequence associated with Gram-negative bacteria (LPS) [112], but it has been shown to increase in Gram-positive associated IMI [15].

#### Genes negatively-associated with TNF

As discussed above, several genes involved in milk fat synthesis were down-regulated in *S. uberis*-infected quarters. Network analysis by IPA indicated that the products of *CD36, GPAM, FABP4, LPIN1, LPL*, and *SCD*, known to be involved in milk fat synthesis [51], were negatively-associated with the expression of *TNF*. It has been demonstrated that TNF reduces expression of *LPL *in rat adipocytes [113]; *GPAM *in mouse adipocytes [114], and *CD36, FABP4*, and *SCD *in adipocytes from human, mouse, or rat [115-117]. Most researchers examining gene expression responses after IMI challenge have primarily focused on genes involved with the immune response, and very few studies [8] have examined large-scale gene expression profiles in the mammary gland during an IMI challenge with *S. uberis*. This research provides evidence of a role for TNF in modulation of milk fat synthesis in the mammary gland during an IMI.

## Conclusion

Our study indicated that IMI challenge with *S. uberis *(strain O140J) elicited a strong transcriptomic response, leading to an overall up-regulation of genes involved in the innate immune response. Results provided additional information into the early response factors associated with the innate immune response to *S. uberis *infection. Although the degree of down-regulation among DEG during IMI challenge was not as marked (<2.5-fold change in expression), it was interesting that the majority of these genes were associated with lipid metabolism and, particularly, milk fat synthesis. Pathway analysis suggested an inhibitory effect of IMI on LXR and PPAR signaling (most likely PPARγ). The latter may provide a mechanistic explanation for the inverse relationship between immune response and milk fat synthesis. This finding deserves more attention due to the possibility of manipulating PPAR signaling through diet.

Milk composition analysis would be useful in the future to relate with mammary gene expression changes during an IMI. The growing amount of information regarding differences in mammary response to major mastitis-causing pathogens such as *E. coli, Staph. aureus*, and *S. uberis *has provided researchers with new insights into the transcriptomic mechanisms involved in immune response and metabolism during an IMI. Mechanisms involved in the immune response to these invading microorganisms warrant further investigation.

## Methods

All procedures involving animals received approval from the Institutional Animal Care and Use Committee at the University of Illinois at Urbana-Champaign (protocol 05179). Details of animal management and preparation of bacterial inoculum are published elsewhere [14]. Briefly, 10 multiparous Holstein cows in mid-lactation were used for this study. To be eligible, cows must have exhibited positive energy balance for > 2 consecutive weeks with composite milk SCC < 200,000 cells/mL, and cows must not have been treated for clinical signs of mastitis or any other disease during early lactation. Since composite rather than quarter foremilk samples were collected, all quarters from all cows must have been bacteriologically negative to confirm that no quarters were sub-clinically infected with an invading pathogen. Eligible cows were paired based on parity, days in milk and milk yield. Cows were housed and fed in individual tie-stalls, had free access to water, and were milked twice daily at 0500 and 1700 h. Cows averaged 39.2 ± 7.4 kg milk/d and were 77 ± 12 days in milk at the start of the trial. A primary objective was to evaluate the effect of negative energy balance (NEB) on immune response reported elsewhere [14]. At ~77 days in milk, half of the cows (n = 5) were feed-restricted to 60% of calculated net energy for lactation requirements to induce NEB. Feed restriction lasted 7 days. Control cows (n = 5) were fed the same diet ad libitum (i.e., positive energy balance; PEB).

Prior to inoculation, a 10-μL loopful of *S. uberis *colonies (strain O140J; provided by J. Hogan; The Ohio State University, Wooster) was incubated in 100 mL of Todd-Hewitt broth for 6 h at 37°C. Following incubation, the broth culture was diluted in sterile Mammalian Ringer's Solution (Electron Microscopy Sciences, Hatfield, PA) to yield ca. 5,000 cfu in a 2-mL volume (i.e., 2,500 cfu/mL). Following the afternoon milking on day 5 (132 h; h = 0 of infection) of feed restriction, 2 mL of inoculum containing *S. uberis *was infused into one rear quarter of each cow via a sterile disposable syringe fitted with a sterile teat cannula using the full insertion infusion method. Prior to inoculation, challenged teats were rigoursly cleaned with cotton balls containing 70% isopropyl alcohol. Immediately following inoculation, all teats were immersed in a postmilking teat disinfectant containing 1% iodine with lanolin. Systemic and local inflammatory indicators were used to monitor the clinical response to intramammary *S. uberis *challenge as described by [14]. Briefly, rectal temperature, heart rate, respiration rate, and fecal scores were evaluated at 0, 3, 6, 12, 14, 16, 18, 20, 24, 30, 36, 42, and 48 hours post-challenge. Based on previous experience [17], peak clinical signs of *S. uberis *inoculation were expected at 24 to 36 hours post-inoculation. At 20 h post-inoculation, and before peak clinical signs, both the *S. uberis *infected (i.e., **YES**) and non-infected (i.e., **NO**) rear quarters were biopsied for RNA extraction and microarray analysis. Duplicate samples of quarter foremilk were aseptically collected for bacteriological examination and SCC before feed restriction and immediately before IMI challenge. In addition, samples were collected at 12, 20, 24, 30, and 36 h post-challenge to confirm infection by quantifying bacterial and SCC concentrations. The SCC was determined using infrared procedures (FOSS 4000, Dairy Lab Services, Inc., Dubuque, IA). Foremilk samples for culture were collected aseptically according to National Mastitis Council recommendations [118].

Details of mammary biopsy, RNA isolation, microarray procedure, primer design and qPCR analysis are found in Additional File 1.

### Statistical analysis

#### Microarrays

Oligonucleotides that were flagged with "-100" by GenePix were removed from the analysis, and the remaining data were normalized to control for dye effects using the median of control elements on the microarray. In a subsequent normalization step, the log_2 _normalized ratio of mammary versus reference (i.e., RNA mixture of different tissues including mammary) signal intensities were adjusted for global dye and microarray effects and normalized by Lowess. A mixed-effects model was then fitted to the adjusted ratios (mammary/reference) using Proc MIXED [119]. The model consisted of treatment (**TRT**; NEB and PEB), infection (**INF**; YES and NO), and the TRT × INF interaction. YES identifies mammary quarters inoculated with 5,000 cfu of *S. uberis*; and NO identifies contralateral rear control quarters (i.e., non-inoculated). The fixed effect was dye with cow and microarray as random effects. Statistical significance probability values for TRT, INF and TRT × INF effects were adjusted for the number of comparisons using Benjamini and Hochberg's FDR. For this paper, the effect of INF, regardless of TRT [14], will be discussed. Differentially expressed genes were based on FDR *P*-value ≤ 0.06 which corresponded to an unadjusted *P *≤ 0.01. Fold change was presented as the backtransformed LSMeans (i.e., adjusted log_2 _normalized ratios of mammary versus reference) of the infected versus non-infected quarters.

#### qPCR

After normalization with internal control genes (**ICG**, see above), data were analyzed using the MIXED procedure of SAS with a random effect of pair within block (day of inoculation). Class variables included cow, TRT, INF, pair, and block. The model included TRT, INF and the TRT × INF. Statistical differences were declared as significant and highly significant at *P *< 0.05 and *P *< 0.01. Trends towards significance are discussed at *P *< 0.10. Relative expression values are presented as least square means (**LSM**). The fold change was presented as the LSMeans of the infected versus non-infected quarters.

### Microarray data

The microarray data files discussed in this publication have been deposited in NCBI's Gene Expression Omnibus (GEO; http://www.ncbi.nlm.nih.gov/geo/) and are accessible through GEO series accession number [GSE15344].

### Data mining

Networks, functions, and pathways analyses were generated using IPA (Ingenuity Systems, http://www.ingenuity.com, Redwood City, CA) which assists with microarray data interpretation via grouping DEG into known functions, pathways, and networks based primarily on human and rodent studies. In addition, data were analyzed using GO by means of GeneSpring GX7 (Agilent Technologies, Santa Clara, CA).

### Approach used in IPA

The 2,102 oligos (with FDR ≤ 0.06) with their associated annotation (when present) and the LSmean (after back-transformation) were uploaded into IPA. Data from qPCR analysis instead of microarray were used for those genes verified. Because we uploaded data from part of the oligonucleotides present on the microarray (i.e., those with FDR ≤ 0.06), the IPA Knowledge Base was used as a reference set for statistical analysis of enriched functions/pathways. This approach suffers from the biases towards overrepresented functions in the bovine oligonucleotide microarray platform. Each annotated gene was mapped to its corresponding gene object in the IPA Knowledge Base. The 2,102 DEG were run without fold-change cut-off or with 1.5-fold change cut-off (a total of 173 genes passed this last criterion). The latter was done with the purpose of identifying highly affected functions. For both datasets, several analyses were run:

- *Functional Analysis*. The functional analysis in IPA identified the biological functions that were most significant to the data set. To minimize false positives among significantly-enriched functions an FDR ≤ 0.000001 (-log *P*-value = 6.0) was used to determine the probability that each biological function assigned to that data set was due to chance alone.

- *Network Generation*. It was conducted only with the 158 DEG with a ≥ 1.5-fold cut-off that were eligible to generate a network. See the above sections for greater description.

- *Canonical Pathway Analysis*: Canonical pathway analysis identified the pathways from the IPA library that were most significant to the data set. Genes from the data set that were associated with a canonical pathway in the IPA Knowledge Base were considered for the analysis. The significance of the association between the data set and the canonical pathway was measured in 2 ways: 1) a ratio of the DEG that mapped to the pathway divided by the total number of genes that mapped to the canonical pathway; 2) an FDR ≤ 0.0005 to calculate a *P*-value determining the probability that the association between the DEG and the signaling canonical pathway was explained by chance alone. For metabolic pathways a less stringent approach (FDR ≤ 0.06) was used because no metabolic pathways were present among the canonical pathways at an FDR corrected *P*-value ≤ 0.0005.

### Criteria used to interpret the IPA functional analysis

These criteria are valid only for the analysis of the 2,102 DEG. The description of the functions in IPA was a consideration of the response of the genes (up- or down-regulated) and the "effect on function" feature in IPA. The final evaluation on the effect on any particular function was an extrapolation of the ensemble following these criteria (see Additional Files 3, 4, 5, 6, 7, 8 and 9):

- when a function in IPA "effect of function" had a number of genes in "increase/decrease function" that was <10% higher from those in "decrease/increase function" including genes in "affect function" which evidently induce or inhibit the function (assessed by carefully considering the IPA links which include IPA descriptions or the original papers for those functions) the functions were considered to be in equilibrium or not having a net effect (or not evident net effect). Further, even though the function was significantly enriched with DEG, a final judgment of a biological outcome was not feasible, thus the function was considered in equilibrium (denoted by ↔).

- when a function in IPA "effect of function" had a number of genes in "increase/decrease function" that was ≥ 10% higher from those in "decrease/increase function" including genes in "affect function", which evidently induce or inhibit the function (as reported above), the function "tends to increase/decrease (or induced/inhibited)" which for simplicity was denoted with arrows (tendency to induce or increase = ↑; tendency to inhibit or decrease = ↓);

- when the number of genes which increase/induce or decrease/inhibit the function was ≥ 100% more (or ≥ 2-fold) compared to decrease/inhibit or increase/induce, the function was considered to be evidently induced or inhibited (simple arrows ⇑ or ⇓);

- when all, or nearly all, the genes found in increase/induce or decrease/inhibit function or the analysis of "affect function" stated that they were involved in inducing or inhibiting the function, the function was considered to be completely induced or inhibited (⇑⇑ or ⇓⇓);

- genes which were up-regulated and were found in "decrease function" were considered to actively decrease or inhibit the function;

- genes which were down-regulated and were found in "decrease function" were considered to decrease the function and also to allow the function to take place;

- genes which were up-regulated and were associated with "increase function" were considered to increase or induce the function;

- genes which were down-regulated and were associated with "increase function" were considered as failing to increase or induce the function;

- the final evaluation on the state of a particular function was a sum of all up- and down-regulated genes.

### GO analyses

This analysis was performed by means of GeneSpring GX7 with the annotation updated on March 3, 2009 by the automatic annotation feature in GeneSpring GX7 using GeneBank accession numbers. The updated GO had 7,710 annotated out of 13,257 total oligos in Biological process (Bp), 7,765 in Cellular component (Cc), and 8,327 in Molecular function (Mf) oligos. The analysis was run for the overall DEG (2,102 oligos, 1,359 annotated for Bp, 1,365 for Cc, and 1,452 for Mf; Additional File 15), for the overall up-regulated DEG (1,082 oligos, 738 annotated for Bp, 731 for Cc, and 776 for Mf), overall down-regulated DEG (1,020, 621 annotated for Bp, 634 for Cc, and 676 for Mf); DEG with ≥ 1.5-fold change (173 oligos, 130 annotated for Bp, 128 for Cc, and 132 for Mf; Additional File 16), up-regulated DEG ≥ 1.5-fold change (130 oligos, 99 annotated for Bp, 101 for Cc, and 101 for Mf), and down-regulated DEG ≥ 1.5-fold change (43 oligos, 31 annotated for Bp, 27 for Cc, and 31 for Mf). The *P*-value was set at 0.05. Results from each GO category and from each list was saved as a text file, and formatted and processed with Excel (see Additional Files 15 and 16). All GO results with a *P*-value ≤ 0.05 are available in Additional Files 15 (i.e., all DEG) and 16 (DEG with ≥ 1.5-fold).

### Criteria used to interpret the GO categories and build GO figures

The GO analysis provided a *P*-value and the number of genes in each sub-category within Bp, Mf, or Cc. The most representative list is that using the overall DEG (2,102 oligos), which simultaneously considered all genes/functions which were affected regardless of the direction of the change (see Additional Files 15 and 16). This allowed visualization of the enrichment of GO categories but did not facilitate an interpretation of the biological effect (i.e., inhibition or activation). The separate analysis of up- and down-regulated DEG was performed with the purpose of overcoming this limitation. Because all genes in each specific list had the same direction the interpretation was facilitated. However, it is important to note that use of only the separate analysis could be tricky because there are GO categories which can be enriched equally in up- and down-regulated DEG, thus precluding a conclusion except on the evidence of the enrichment. Using Microsoft Excel software we clustered all the GO categories significantly enriched in the overall gene list, ⇑ genes and ⇓ genes simultaneously, and by means of Pivot table in Excel, we compared the simultaneous enrichment of each specific category in overall, up-, and down-regulated DEG. When the significantly-enriched categories in the overall DEG list were enriched in up- and down-regulated DEG, we concluded that the category was induced (if the significant enrichment was present in up-regulated DEG) or inhibited (if the significant enrichment was present in down-regulated DEG). When the enriched category in the overall DEG list was present in both up- and down-regulated DEG we concluded that the category was induced if the number of up-regulated DEG was at least 30% greater than in down-regulated DEG and inhibited when vice versa. If a category in the overall DEG list was also present in up- and down-regulated DEG and the aforementioned criteria was not satisfied or the category was absent from both up- and down-regulated DEG, we concluded that the category was enriched but without a clear directional (up or down) effect.

## Authors' contributions

KMM participated in the design of the study, organized the research trial, performed mammary tissue biopsies, performed microarray and qPCR analyses, performed qPCR statistical analysis, and wrote the manuscript. JKD helped with the design of the study and helped draft the manuscript. DEM helped with the design of the study and helped write the manuscript. MB participated in qPCR analysis, identification of ICG, primer design, helped with data mining using IPA and GO, and helped write the manuscript. SLR performed microarray statistical analysis. REE and HAL contributed new reagents and tools. JJL conceived and designed the study and wrote the manuscript. All authors read and approved the final manuscript.

## Supplementary Material

Additional file 1 **Methods and Figures S1 and S2**. The file contains additional results including **Table S1 **titled 'Quantitative PCR (qPCR) and microarray gene expression results due to an intramammary infection'; **Figure S1 **titled 'Individual quarter milk SCC (A) and shedding of *S. uberis *(B) from all 10 cows before (hour 0), after (hour 12) and prior to mammary biopsy (hour 20) for gene expression prolifing'; and **Figure S2 **titled 'Gene expression changes of specific neutrophil and macrophage markers (from http://www.antibodybeyond.com/index.htm) in mammary tissue of Holstein cows treated with *S. uberis *(strain O140J) (YES) or control (NO) quarters'. This file also contains additional materials and methods. RNA isolation, primer design and testing, quantitative PCR and identification of internal controls accompanied by 4 tables: **Table S2 **titled 'GenBank accession number, hybridization position, sequence, amplicon size, and source of primers for Bos taurus used to analyze gene expression by qPCR. List also includes primers for internal control gene'; **Table S3 **titled 'Sequencing results obtained from qPCR product of Bos taurus specific'; **Table S4 **titled 'Sequencing results of genes using BLASTN from NCBI against nucleotide collection with total score'; and **Table S5 **titled 'qPCR performance including slope and coefficient of determination of the standard curve (R^2^), efficiency (E)^2^, and median cycle threshold (Ct) of the measured transcripts'. Click here for file

Additional file 2 **All 2,102 DEG**. The file contains the complete list of DEG based on microarray analysis that includes statistical *P*-values and fold-change of expression for each gene due to IMI with *S. uberis*. Click here for file

Additional file 3 **Functions UP and DOWN all DEG**. Overall functions up and down-regulated during IMI with regards to all DEG (n = 2,102) using IPA Knowledge Base (Ingenuity Systems, Inc.). Click here for file

Additional file 4 **Functions UP all DEG**. Functions up-regulated during IMI with regards to all DEG (n = 2,102) using IPA Knowledge Base. Click here for file

Additional file 5 **Functions DOWN all DEG**. Functions down-regulated during IMI with regards to all DEG (n = 2,102) using IPA Knowledge Base. Click here for file

Additional file 6 **Metabolic pathways all DEG**. Overall canonical metabolic pathways most enriched during IMI with regards to all DEG (n = 2,102) using IPA Knowledge Base. Click here for file

Additional file 7 **Signaling pathways all DEG**. Overall canonical signaling pathways most enriched during IMI with regards to all DEG (n = 2,102) using IPA Knowledge Base. File includes glucocorticoid signaling. Click here for file

Additional file 8 **Metabolic and signaling pathways UP all DEG**. Canonical metabolic and signaling pathways up-regulated during IMI with regards to all DEG (n = 2,102) using IPA Knowledge Base. Click here for file

Additional file 9 **Metabolic and signaling pathways DOWN all DEG**. Canonical metabolic and signaling pathways down-regulated during IMI with regards to all DEG (n = 2,102) using IPA Knowledge Base. Click here for file

Additional file 10 **Functions UP of DEG with 1.5 fold change or greater**. Functions up-regulated during IMI for DEG with expression > 1.5-fold using IPA Knowledge Base. Click here for file

Additional file 11 **Functions DOWN of DEG with 1.5 fold change or greater**. Functions down-regulated during IMI for DEG with expression > 1.5-fold using IPA Knowledge Base. Click here for file

Additional file 12 **Metabolic and signaling pathways UP and DOWN of DEG with 1.5 fold change or greater**. Overall canonical metabolic and signaling pathways up- or down-regulated during IMI for DEG with expression > 1.5-fold using IPA Knowledge Base. Click here for file

Additional file 13 **All 19 networks of DEG with 1.5 fold change or greater**. All networks (n = 19; 158 DEG) within IPA analyses associated with IMI for DEG with expression > 1.5-fold using IPA Knowledge Base. Click here for file

Additional file 14 **Top 5 networks of DEG with 1.5 fold change or greater**. Top 5 networks (100 DEG) within IPA analyses associated with IMI for DEG with expression > 1.5-fold using IPA Knowledge Base. Click here for file

Additional file 15 **GO analysis all DEG**. This file contains results from GO analysis of the 2,102 DEG classified according to biological process, molecular function, and cellular components. Analysis of GO was conducted in GeneSpring GX 7.0 (Agilent Technologies). Click here for file

Additional file 16 **GO analysis of DEG with 1.5 fold change or greater**. This file contains results from GO analysis of the DEG with expression >1.5-fold according to biological process, molecular function, and cellular components. Analysis of GO was conducted in GeneSpring GX 7.0 (Agilent Technologies). Click here for file
