# Supplementary material for: Gene network and pathway analysis of bovine mammary tissue challenged with Streptococcus uberis reveals induction of cell proliferation and inhibition of PPARγ signaling as potential mechanism for the negative relationships between immune response and lipid metabolism
Source: BMC Genomics. 2009 Nov 19;10:542. doi: 10.1186/1471-2164-10-542 (PMC2784807; doi:10.1186/1471-2164-10-542)
Supplement: Additional file 1 — Methods and Figures S1 and S2. The file contains additional results including Table S1 titled 'Quantitative PCR (qPCR) and microarray gene expression results due to an intramammary infection'; Figure S1 titled 'Individual quarter milk SCC (A) and shedding of S. uberis (B) from all 10 cows before (hour 0), after (hour 12) and prior to mammary biopsy (hour 20) for gene expression prolifing'; and Figure S2 titled 'Gene expression changes of specific neutrophil and macrophage markers (from http://www.antibodybeyond.com/index.htm) in mammary tissue of Holstein cows treated with S. uberis (strain O140J) (YES) or control (NO) quarters'. This file also contains additional materials and methods. RNA isolation, primer design and testing, quantitative PCR and identification of internal controls accompanied by 4 tables: Table S2 titled 'GenBank accession number, hybridization position, sequence, amplicon size, and source of primers for Bos taurus used to analyze gene expression by qPCR. List also includes primers for internal control gene'; Table S3 titled 'Sequencing results obtained from qPCR product of Bos taurus specific'; Table S4 titled 'Sequencing results of genes using BLASTN from NCBI against nucleotide collection with total score'; and Table S5 titled 'qPCR performance including slope and coefficient of determination of the standard curve (R2), efficiency (E)2, and median cycle threshold (Ct) of the measured transcripts'. [file 1471-2164-10-542-S1.DOC]

**Additional File 1**

**SUPPLEMENTARY MATERIALS AND METHODS**

***Biopsy procedure.*** Surgeries were performed under aseptic field conditions. Cows were restrained in a squeeze chute to minimize movement. Cows were sedated using intravenous administration of xylazine HCl (35 µg/kg BW; Phoenix Pharmaceuticals, St. Joseph, MO). The hair around the tip of the tail was clipped and the tail was tied to prevent contamination of the surgical site. The biopsy site was carefully selected to avoid subcutaneous blood vessels as well as the cisternal region. An area of skin (10 cm^2^) on the rear quarter was clipped closely, and then washed and sterilized with iodine surgical scrub and 70% ethanol. For local anesthesia, lidocaine HCl (5 mL; Phoenix Pharmaceuticals, St. Joseph, MO) was administered subcutaneously. The biopsy site was then washed an additional 3X as described above. After washing, a 5-cm incision was made through the skin and underlying fascia to the point where the mammary gland capsule was visible. The biopsy trocar was attached to a high-speed (16 Volts) cordless drill. The trocar consisted of a stainless steel cannula of 90 mm in length with a 6-mm diameter containing a retractable blade at the cutting edge of the cannula to sever the core of the tissue once it was cut. Tissue (≥ 0.5 g) was blotted with sterile gauze to remove any visible milk secretions and any visible connective tissue was removed. Mammary tissue was then frozen immediately in liquid nitrogen and stored until isolation of RNA. Once the tissue was collected, pressure was applied to the incision area until the cessation of bleeding. The skin incision was closed with Michel wound clips (11 mm; Down Surgical, Mississauga, ON, Canada) and a coating of Prodine (Phoenix Pharmaceutical, Inc., St. Joseph, MO) iodine ointment was applied to the surgical site.

***RNA isolation.*** Total RNA was isolated from mammary tissue using TRIzol® Reagent (Invitrogen, Carlsbad, CA). Mammary tissue was homogenized in TRIzol with 1 µL linear acrylamide (Ambion, Inc., Austin, TX) using a Tissue-Tearor™ (BioSpec Products, Inc.) homogenizer at maximum speed. Upon centrifugation, total RNA was separated with chloroform followed by acid phenol:chloroform (Ambion, Inc., Austin, TX) to remove DNA. Total RNA was then precipitated with isopropanol and the RNA pellet was cleaned with 75% ethanol prior to reconstitution in RNA storage buffer (Ambion, Inc., Austin, TX) and storage at -80 °C. Total RNA was cleaned using RNeasy mini kit columns and residual DNA removed using the RNase-Free DNase Set (Qiagen, Valencia, CA) prior to microarray or qPCR. RNA quality was assessed using a 2100 Bioanalyzer (Agilent Tenchnologies). Purity of RNA were assessed by ration of optical density OD_260mm_ (oligonucleotides assorbance) /OD_280mm_ (protein assorbance) using NanoDrop ND-1000 (NanoDrop Technologies, Rockland, DE). The ratio of the sample was > 1.7.

***Microarray analysis*.** We used a bovine oligoarray with >13,000 annotated genes that was previously described [1, 2]. A condensed platform (processed/normalized data averaging the duplicate features of platform GPL2853) was used for this paper and is publicly accessible in the National Center for Biotechnology Information (NCBI) Gene Expression Omnibus (GEO) database (GPL8776). Arrays were run in a dye-swap reference design. The reference was made by pooling RNA from several bovine tissues. The cDNA was obtained by RT-PCR in a 30 μL reaction adding 10 to 15 μg RNA, 2 µl of random hexamer primers (3 mg/ml; Invitrogen Corp., CA) and 1 µg oligo dT18 (Operon Biotechnologies, AL), and DNase-RNase free water to a volume of 17.78 μL. The mixture was incubated at 65°C for 5 min and kept on ice for 3 min. In the mixture were added 12.2 μL solution composed of 6 μL 5X First-Strand Buffer, 3 μL 0.1 M DTT, 0.6 μL 100 mM dNTP mix (Invitrogen Corp., CA), 0.12 μL of 50 mM 5-(3-aminoallyl)-dUTP (Ambion, CA), 2 μL (100 U) of SuperScript^TM^ III RT (Invitrogen Corp., CA), and 0.5 μL of RNase Inhibitor (Promega, WI). The reaction was performed at 23°C for 1 min and 46°C for 9 h. The cDNA obtained was then treated with 10 µl 1M NaOH, and incubate for 15 min at 65°C to remove residual RNA. Solution was neutralized by adding 10 µl 1M HCl. The unincorporated 5-(3-aminoallyl)-dUTP and free amines where removed using Qiagen PCR Purification Kit (Qiagen, Germany). Clean cDNA was dry and resuspended in 4.5 µL 0.1 M Na_2_CO_3_ buffer (pH 9.0) and 4.5 µL of Amersham CyDye™ fluorescent dyes diluted in 60 µl of DMSO (Cy3 or Cy5; GE Healthcare, USA). Binding of Cy dyes with 5-(3-aminoallyl)-dUTP incorporate in cDNA was obtained by incubation at room temperature for 1h. The unbound dyes were removed using Qiagen PCR Purification Kit (Qiagen, Germany) and clean labelled cDNA was measured by mean of NanoDrop ND-1000 spectrophotometer ([www.nanodrop.com](http://www.nanodrop.com/)). Critical for accurate microarray analysis is a correct competition between sample and reference which require adding in the slide the same amount of the two labeled cDNA. For this, prior hybridization, an equivalent amount of reference and sample were calculated and placed in two separate 1.5 mL microfuge tubes and vacuum-dried in dark.

***Microarray hybridization and image acquisition***. Prior hybridization slides were re-hydrated, treated at the UV cross linker, washed with 0.2% SDS solution, deeply rinsed with purified water to remove all not-bound oligos, and pre-hybridized using a solution containing 1% albumin, 5 × SCC, and 0.1% SDS at 42 C° for ≥45 min with the purpose to decrease background. After pre-hybridization slides were rinsed with abundant purified water and immerged in isopropanol for ~10s and spin dried. Dried slides were immediately hybridized according to a dye-swap-reference design (i.e. each sample was labelled twice using the two dyes and hybridized in each slide with the reference labelled with the opposite dye). Therefore, a total of 40 slides (i.e. 10 cows × 2 quarters per cow × 2 dyes per quarter) were hybridized to duplicate slides and repeated with reverse labeling for a total of 2 slides per quarter per cow.

Labelled cDNA of the sample was re-hydrated with 80µl of hybridization buffer #1 (Ambion) and mixed thoroughly. The same solution was transferred in the reference with opposite dye and mixed in order to obtain a homogenous solution of the two labeled cDNA. Before hybridization into the slide the labeled cDNA resuspension of the sample + reference was incubated at 90-95°C for ca. 3 min to allow for cDNA denaturation with the purpose to increase the efficiency of binding with oligos into the slide.

The hybridization was carried out using humidified slide chamber (Corning) with cover slips (LifterSlip, Thermo Scientific) at 42 C° for about 40 hours in dark. After hybridization the slides were removed from the chamber and washed by 5 min agitation for 3 times in temporal order: 1×SSC and 0.2% SDS solution preheated at 42°C, 0.1×SSC and 0.2% SDS, solution, and 0.1×SSC solution. Finally, slides were spin dried and inserted in a 50 mL tubes were Argon gas was added to preserve dye from degradation. Arrays were scanned with a ScanArray 4000 (GSI-Lumonics, Billerica, MA) dual-laser confocal scanner and images were processed and edited using GenePix 6.0 (Axon Instruments). Array quality was assessed using homemade parser software written in Perl language as previously described [3]. Spots in the slide were considered good if the median intensity was ≥3×standard deviation above median background for each channel (i.e., dye). Spots were flagged present when both dyes passed the criteria, marginal if only one dye passed the criteria, absent when both dyes failed to pass the criteria. In the analysis both present and marginal spots were used.

***Primer design and testing for qPCR.*** Primers were designed as previously described [3]. Briefly, Primer Express software version 3.0 (Applied Biosystems, Foster City, CA) was used with defaults features except min amplicon length to 100. Primers were aligned using publicly available databases including BLASTN at NCBI and UCSC’s Cow (*Bos taurus*) Genome Browser Gateway (http://genome.ucsc.edu/cgi-gin/ghGateway). Primers were first tested using the SYBR Green protocol as describe below without the dissociation protocol. Five μL of the PCR products were run on a 2% agarose gel (Invitrogen, Carlsbad, CA) stained with ethidium bromide to assess presence of the product to a expected size and presence of primer-dimer, the rest was purified using Qiaquick PCR purification kit (Qiagen, Valencia, CA) and sent to Core DNA Sequencing Facility of the Roy J. Carver Biotechnology Center at the University of Illinois, Urbana, for sequencing. Table S2 shows the list of all primers designed and used for qPCR analysis in this study. All sequences were confirmed using NCBI and UCSC’s cow Genome Browser Gateway and are shown in Tables S3 and S4.

***Identification of internal control genes.*** In order to verify microarray results from present and other studies [4-6] a subset of genes (*TNF, BAX, FOS, SOD1, SAA3, IL6, LPL, SCD, LPIN1, SCD, LALBA, CSN3,* and *CD36*) was initially analyzed via qPCR using *GTP, UXT*, and *MRPL39* as internal control genes (**ICG**). Among several genes tested, these ICG were found to be highly stable (i.e., normalization factor stability <0.10) [3], and have been subsequently used in other studies as ICG of bovine mammary gland [7]. Despite their suitability as ICG, it was deemed necessary to perform a more detailed analysis of potential ICG. Detailed description for the selection criteria of ICG for qPCR analysis was previously described [8]. Briefly, GeneSpring GX software (Agilent Technologies, Santa Clara, CA) was used to identify mammary tissue gene expression stability among all samples, i.e., INF (YES and NO) and TRT (NEB and PEB). Genes with a constant normalized expression ratio (~1.0; sample/reference) and with the greatest abundance on microarrays (i.e., 20 out of 20 arrays, 10 cows × 2 quarters, and >100 relative fluroscence unit) were further used for ICG co-regulation evaluation using Ingenuity Pathway Analysis® software (**IPA**; Redwood City, CA). Co-regulation is described as common upstream regulatory factors or direct regulation of transcript expression between gene products. Genes without known co-regulation from the selected genes or transcription factors were further used for ICG evaluation through geNorm [9]. These included *MMRN2*, *DNASE1*, *NENF*, and *GRHL1*. geNorm determines both the optimal number of ICG to obtain a reliable normalization factor (**NF**) and calculates the NF. The optimal number of ICG were determined by assessment of the pairwise variation V (V_n/n+1_) between the normalization factors NF_n_ and NF_n+1_. Due to the very high stability of NF using 3 genes (V = 0.008) *DNASE1*, *NENF*, and *GRHL1* were used for calculation of NF. The V of NF using those 3 genes (V = 0.008) proved to be even more stable than those used initially (V = 0.094).

***qPCR*.** For cDNA synthesis, 100 ng of purified (described above) RNA sample was added with 1 µg dT18 (Operon Biotechnologies Inc., Germantown, MD), 1 µL 10 mM dNTP mix (Invitrogen, Carlsbad, CA), 1 µL Random Primers (Invitrogen, Carlsbad, CA), and 7 µL DNase/RNase free water. The mixture was incubated for 5 min at 65°C using an Eppendorf Mastercycler Gradient and then placed on ice for 3 min. A total of 9 µL of Master Mix consisting of 4.0 µL 5X First-Strand Buffer (Invitrogen, Carlsbad, CA), 1 µL 0.1 M DTT, 0.25 µL of SuperScript^TM^ III reverse transcription (Invitrogen, Carlsbad, CA), 0.25 µL of RNase Inhibitor (Promega, Madison, WI) and 3.5 µL of DNase/RNase free water was then added. The mixture was incubated for 5 min at 25°C followed by 60 min at 50°C and then for 15 min at 70°C. The cDNA was then diluted 1:4 with DNase/RNase free water. A combination of 4 µL of diluted cDNA with 6 µL of 1× SYBR Green master mix (Applied Biosystems, Foster City, CA), 0.4 µL each of 10 µM forward and reverse primers, and 0.2 µL of DNase/RNase free water were added to each well of a MicroAmp Optical 384-Well Reaction Plate (Applied Biosystems). All sample reactions and a 6-point standard curve (see below) were run in triplicate. The reactions were performed in an ABI Prism 7900 HT SDS instrument (Applied Biosystems) under the following conditions: 2 min at 50°C, 10 min at 95°C, 40 cycles of 15 s at 95°C, and 1 min at 60°C. During the last cycle (95°C for 15 s, 65°C for 15 s), a dissociation protocol was used to specify the amplicons, the presence of a single PCR product. The standard curve was generated by pooling cDNA from all 20 experimental samples (i.e., 10 cows with 2 rear quarter samples each). A 6-point standard curve was generated via serial dilution of the pooled standard using DNase/RNase free water to obtain a 1:5 (v:v) or 20% dilution per point. Table S5 shows the slope and coefficient of determination of the standard curve, efficiency, and median cycle threshold (**Ct**) of the measured transcripts from qPCR SDS documents generated from the ABI Prism 7900 HT SDS instrument.

| **Table S2.**  GenBank accession number, hybridization position, sequence, amplicon size, and source of primers for *Bos taurus* used to analyze gene expression by qPCR. | | | | | |
| --- | --- | --- | --- | --- | --- |
| Accession # | Gene | Primer | Primers (5’- 3’) | Amplicon Size (bp) | Source |
| NM_001076058 | *ACP2* | F.551 | GAGTGTTCAGAATGCACAATTCCT | 100 | This manuscript |
|  |  | R.650 | GAATAGCGTGTCGTAGACATTCCA |  |  |
| BT029909 | *ADFP* | F.139 | TGGTCTCCTCGGCTTACATCA | 81 | [7] |
|  |  | R.219 | TCATGCCCTTCTCTGCCATC |  |  |
| BC114132 | *ADRB2* | F.1181 | CATCAACTCCGCTTTCAATCC | 110 | This manuscript |
|  |  | R.1290 | CATTCCCATAGGCCTTCAATG |  |  |
| NM_001076293 | *ALOX5AP* | F.114 | ACAAGGTGGAGCACGAAAGC | 100 | This manuscript |
|  |  | R.213 | ACACAGTTCTGGTTGGCAGTGT |  |  |
| BT021501 | *ANXA1* | F.737 | GCCCCTATCCTACATTCAATCCA | 120 | This manuscript |
|  |  | R.836 | GCTGTGCATTGTTTCTCTTAGTCAGA |  |  |
| U92569 | *BAX* | F.75 | TTTGCTTCAGGGTTTCATCC | 246 | This manuscript |
|  |  | R.320 | CAGTTGAAGTTGCCGTCAGA |  |  |
| XM 610447.3 | *BCL3* | F.232 | AGATACGCCACTCCACATTGC | 113 | This manuscript |
|  |  | R.344 | GCCGGAGGTTGTTGTAGATATCC |  |  |
| NM_001035293 | *BIRC3* | F.1100 | TGTGAAATGCTTTTGCTGTGATG | 100 | This manuscript |
|  |  | R.1199 | TAAGTACTCACACCGTGGAAACCA |  |  |
| NM_001130761 | *BNBD5* | F.50 | TGTCTGCTGGGTCAGGATTTACT | 90 | This manuscript |
|  |  | R.139 | CAGGGCACGAGATCGGAATA |  |  |
| XM_587039 | *C1QC* | F.413 | GGTGTGGAGGGCAGGTACAA | 100 | This manuscript |
|  |  | R.512 | GGTTGAACTTGACCAGGCTGTT |  |  |
| BC112452 | *C3* | F.746 | TTCCCAGTTTTGAGGTCCAACT | 100 | This manuscript |
|  |  | R.845 | CCGTACAAGAACCTGGCAATG |  |  |
| BT030512 | *CASP8* | F.794 | GAGCCTTGAAAGAGATCCATATGC | 130 | This manuscript |
|  |  | R.923 | TGTGTCAGATGTCTGCAACTCACT |  |  |
| NM_001075921 | *CCR1* | F.1292 | AGGTACCGCAGGGTTCTATGG | 110 | This manuscript |
|  |  | R.1401 | TCCTTGCCTGTGTAATGGACAT |  |  |
| BC148880 | *CD14* | F.1366 | CAGTGCTGGACAGGGAATCC | 100 | This manuscript |
|  |  | R.1465 | CTTCTGCCCCCGTAGTTCTTT |  |  |
| X91503 | *CD36* | F.743 | GTACAGATGCAGCCTCATTTCC | 81 | [7] |
|  |  | R.823 | TGGACCTGCAAATATCAGAGGA |  |  |
| AF004943 | *COX1* | F.132 | CACCAAAGGGAAGAAGCAGTTG | 101 | This manuscript |
|  |  | R.232 | ACATGAGGTTGGTGCCTTGAG |  |  |
| BC102120 | *CSN3* | F.445 | GGCGAGCCTACAAGTACACCTA | 106 | This manuscript |
|  |  | R.550 | GGACTGTGTTGATCTCAGGTGG |  |  |
| BC142349 | *DNASE1* | F.548 | CACTCCACCAAGGTCAAGGAAT | 100 | This manuscript |
|  |  | R.647 | CATCCAGGTAGACATCGTAGAGAGAA |  |  |
| CV983373 | *FOS* | F.580 | TCCATGCGTTTTGCTACATCTC | 101 | This manuscript |
|  |  | R.680 | CGTGAAACACACCAGGCTGT |  |  |
| BC119971 | *GRHL1* | F.1654 | GGTGAAGGGTCCAGCTTGAA | 106 | This manuscript |
|  |  | R.1759 | GCAGCACTCTCTTTGGTTCTTCTA |  |  |
| AK074976 | *GTP* | F.696 | CTTGGAATCCGAGGAGCCA | 101 | [3] |
|  |  | R.796 | CCTGGGATCACCAGAGCTGT |  |  |
| BT021059 | *HLA-DRA* | F.594 | AAGTTCCACTACCTCCCCTTCCT | 110 | This manuscript |
|  |  | R.703 | GGAGCTTCATACTCCCAGTGCTT |  |  |
| BC102105 | *HMOX1* | F.191 | CAGGCGGAGAATGCAGAGTT | 81 | This manuscript |
|  |  | R.270 | GACGCCATCACCAGCTTAAAA |  |  |
| EU276074 | *IL10* | F.429 | GAGAGTCTTCAGTGAGCTCCAAGAG | 100 | This manuscript |
|  |  | R.529 | GCATCTTCGTTGTCATGTAGGTTT |  |  |
| BT021904 | *IL10RB* | F.280 | AAGTATGGTGACCACACCTTGAGA | 107 | This manuscript |
|  |  | R.386 | GGAGGTCCGATAGTGGTGTCAT |  |  |
| BTU42433 | *IL15* | F.141 | AGCAAACTGGCAGTATGTAATAAATAA | 109 | This manuscript |
|  |  | R.249 | GCAATTGGGATGAGCATCACT |  |  |
| EU276078 | *IL18* | F.85 | AATGATGAAGACCTGGAATCAGATC | 110 | This manuscript |
|  |  | R.194 | ACAGGTTGATTTCCCTGGTTAATG |  |  |
| M37211 | *IL1B* | F.30 | ATTCTCTCCAGCCAACCTTCATT | 100 | This manuscript |
|  |  | R.129 | TTCTCGTCACTGTAGTAAGCCATCA |  |  |
| BC112665 | *IL1R2* | F.980 | AGCGCCAGGCATACTCAGAA | 90 | This manuscript |
|  |  | R.1069 | AATCCGTGTGCAAATCCTCTCT |  |  |
| BC134577 | *IL1RN* | F.308 | GCTCAAGTTAGAGGCCGTGAA | 118 | This manuscript |
|  |  | R.425 | GCAGGCAGCTGACTCAAAGC |  |  |
| BC123577 | *IL6* | F.290 | TGAGTGTGAAAGCAGCAAGGA | 101 | [2] |
|  |  | R.390 | TCGCCTGATTGAACCCAGAT |  |  |
| EU276073 | *IL8* | F.102 | TGTGAAGCTGCAGTTCTGTCAA | 130 | This manuscript |
|  |  | R.231 | TTTCACAGTGTGGCCCACTCT |  |  |
| XM_589325 | *INSIG1* | F.438 | AAAGTTAGCAGTCGCGTCGTC | 120 | [10] |
|  |  | R.557 | TTGTGTGGCTCTCCAAGGTGA |  |  |
| XM_590552 | *INSR* | F.245 | CCCTTCGAGAAAGTGGTGAACA | 84 | This manuscript |
|  |  | R.328 | AGCCTGAAGCTCGATGCGATAG |  |  |
| DQ319075 | *IRAK1* | F.950 | CCTCAGCGACTGGACATCCT | 103 | This manuscript |
|  |  | R.1052 | GGACGTTGGAACTCTTGACATCT |  |  |
| XM_001790624 | *JAK1* | F.766 | GCTCAGGGACAGTATGATTTGGT | 100 | This manuscript |
|  |  | R.865 | CCATCCCAAGACACTCGTTCTC |  |  |
| BT025469 | *LALBA* | F.28 | CTCTGCTCCTGGTAGGCATC | 125 | This manuscript |
|  |  | R.152 | ACAGACCCATTCAGGCAAAC |  |  |
| AJ551281 | *LDLR* | F.19 | GTTATCGCCGGAGACATTGTC | 100 | This manuscript |
|  |  | R.118 | GGTACTGGCAGCCACCATTG |  |  |
| DV797268 | *LPIN1* | F.147 | TGGCCACCAGAATAAAGCATG | 101 | [11] |
|  |  | R.247 | GCTGACGCTGGACAACAGG |  |  |
| BC118091 | *LPL* | F.327 | ACACAGCTGAGGACACTTGCC | 101 | [7] |
|  |  | R.427 | GCCATGGATCACCACAAAGG |  |  |
| BC116051 | *LTF* | F.891 | CAAGGCGCAGGAGAAATTTG | 105 | [8] |
|  |  | R.995 | AACCCAAGACCAGAGTCTTTGAA |  |  |
| BC112597 | *LYZ* | F.94 | GAGAGATGTGAGCTTGCCAGAA | 120 | This manuscript |
|  |  | R.213 | TGTAGCTTGTGTGTTGTAATTGCTTTC |  |  |
| BF655477 | *MMRN2* | F.79 | GATCGGAAGCAAGTGGAATCTT | 101 | This manuscript |
|  |  | R.179 | CCTTCTGAAAAGCTGGCGTAGA |  |  |
| NM017446 | *MRPL39* | F.493 | AGGTTCTCTTTTGTTGGCATCC | 101 | [3] |
|  |  | R.593 | TTGGTCAGAGCCCCAGAAGT |  |  |
| BC116106 | *NENF* | F.175 | GAGGAGGAAGATCAGCCCATCT | 107 | This manuscript |
|  |  | R.281 | CCGGTCAGGGCGTTGTAG |  |  |
| DN283323 | *NFKB1A* | F.391 | GGGAGACCTGGCCTTCCTC | 101 | This manuscript |
|  |  | R.491 | CCAGAAGTGCCTCAGCGATT |  |  |
| XM_612999 | *NR3C1* | F.27 | AGCCGGCAGAGCTGATACTC | 134 | This manuscript |
|  |  | R.160 | CCTCCCCTTAGGGTTTTATAGAAGTC |  |  |
| BT030737 | *PLAU* | F.400 | GACTCTCCCACCGTCCTTCTG | 100 | This manuscript |
|  |  | R.499 | GATTGTCTGGGTTCCTGCAATAA |  |  |
| BC142002 | *PLAUR* | F.697 | GCCCACAGGTGTTCTTCTGAA | 100 | This manuscript |
|  |  | R.796 | TTGGGTTCCTCAGTCCTTTAGTG |  |  |
| BC140620 | *PRKCB1* | F.1091 | ACAGAAATTCGAGAGAGCCAAGA | 100 | This manuscript |
|  |  | R.1190 | GTCCCTGTTGCCGTTGTTGT |  |  |
| AF540564 | *SAA3* | F.50 | GGGCATCATTTTCTGCTTCCT | 106 | This manuscript |
|  |  | R.155 | TTGGTAAGCTCTCCACATGTCTTTAG |  |  |
| AY241933 | *SCD* | F.665 | TCCTGTTGTTGTGCTTCATCC | 101 | [7] |
|  |  | R.765 | GGCATAACGGAATAAGGTGGC |  |  |
| BC102257 | *SDHD* | F.254 | TTGCTCCTGGGCCTAATTCC | 100 | This manuscript |
|  |  | R.353 | CAATGCCCCAGTGACTGTGA |  |  |
| NM_174182 | *SELL* | F.588 | CTCTGCTACACAGCTTCTTGTAAACC | 104 | This manuscript |
|  |  | R.691 | CCGTAGTACCCCAAATCACAGTT |  |  |
| BC149300 | *SELP* | F.1931 | TTGCAGACAAAAAGATGATGGAA | 100 | This manuscript |
|  |  | R.2030 | TTAAGGGCTTGGGTCAAACG |  |  |
| AY183452 | *SOCS2* | F.995 | CGGCACTGTTCACCTTTATCTG | 100 | This manuscript |
|  |  | R.1094 | GACGGTGCTGGTACACTTGTTAAT |  |  |
| BC102432 | *SOD1* | F.256 | GGCTGTACCAGTGCAGGTCC | 101 | This manuscript |
|  |  | R.356 | GCTGTCACATTGCCCAGGT |  |  |
| AY183452 | *SOD2* | F.272 | CGGCACTGTTCACCTTTATCTG | 100 | This manuscript |
|  |  | R.371 | GACGGTGCTGGTACACTTGTTAAT |  |  |
| BC109481 | *STAT3* | F.1210 | CCGGTGTCCAGTTCACAACTAA | 100 | This manuscript |
|  |  | R.1309 | TCCCCGGAGTCTTTGTCAAT |  |  |
| EU746459 | *TLR2* | F.88 | TGGGTGGAGAACCTCATGGT | 100 | This manuscript |
|  |  | R.187 | CGATAATCCACTTGCCAGGAA |  |  |
| DQ839567 | *TLR4* | F.85 | TGCGTACAGGTTGTTCCTAACATT | 110 | This manuscript |
|  |  | R.194 | TAGTTAAAGCTCAGGTCCAGCATCT |  |  |
| EU276079 | *TNF* | F.174 | CCAGAGGGAAGAGCAGTCCC | 114 | This manuscript |
|  |  | R.287 | TCGGCTACAACGTGGGCTAC |  |  |
| DQ319074 | *TRAF6* | F.675 | AGAACAGATGCCCAATCACTATGAT | 100 | This manuscript |
|  |  | R.774 | GTGATTCCTCTGCATCTTTTCATG |  |  |
| BQ676558 | *UXT* | F.323 | TGTGGCCCTTGGATATGGTT | 101 | [3] |
|  |  | R.423 | GGTTGTCGCTGAGCTCTGTG |  |  |
| AJ609502 | *VLDLR* | F.68 | GCCCAGAACAGTGCCATATGA | 103 | [7] |
|  |  | R.200 | TTTTCACCATCACACCGCC |  |  |

| **Table S3.** Sequencing results obtained from qPCR product of *Bos taurus* specific primers. | |
| --- | --- |
| Gene | Sequence |
| *ACP2* | CATGAGACGGGGCTCACGGACCTGTCCCTGGAGACTGTCTGGAATGTCTACGACACGCTATTCA |
| *ADRB2* | CCATGTCGGACAGATTTCAGGATTGCCTTCCAGGAGCTTCTCTGCCTGCGCAGGTCTTCATTGAAGGCCTATGGGAATGA |
| *ALOX5AP* | CGGGACTCCAGAGGACTGGGACGCTGGCCTTTGAGCGGGTCTACACTGCCAACCAGAACTGTGATAATTTT |
| *ANXA1* | TGAGGCCTTGCCAAGCATCCGTTAGAGGGTGGTGGATGAAGCAACCATCATTGAAATTCTGACTAAGAGAAACAATGCACAGCAA |
| *BCL3* | CGGGCACTGCCGCTGTGCTCGCCTGGGTCAACCTCTTCCAGCATGGAGGCCGGGAACTGGATATCTACAACAACCTCCGGCAA |
| *BIRC3* | TTGGGAATCCTGGAGATGATCCATGGGTGGAACCATGCCAAGTGGTTTCCACGGTGTGAGTACCTTAA |
| *BNBD-5* | GCGTAGAATCCTCAAAGCTGCCGTTGGAATATGGGTGTCTGTATTCCGATCTCGTGCCCTGAAA |
| *C1QC* | GCAGTCAGTGTTCTCAGTCACACGGCAGACTGTCCAGTTCCCGGAGGCCAACAGCCTGGTCAAGTTCAACCAT |
| *C3* | TTGATGACCCAGATGGCCTGAAGGTCAACATCATTGCCAGGTTCTTGTACGGA |
| *CASP8* | ATTAGTATGTCACATCGCTTCCAGAGGAGGGCTACTCTAAGATGCCGGCCATGTCAGACTCTCGCAGAACAGGACAGTGAGTTGCAGACATCTGACACAA |
| *CCR1* | CGTCTTCGTACTAGGTNTCGCTGCTTCCTCTACGAGGAGGCAGGAGAGGGTCAACTCCATGTCCATTACACAGGCAAGGAA |
| *CD14* | CCTCCTAACTTGCTAGAAACCATACCCGCCCTACCATAGAAAAGAACTACGGGGGCAGAAGATTTT |
| *COX-1* | ATCTGAGTCGTCGCTTCCTGCTCAGGAGGAAGTTCATCCCCGACCCTCAAGGCACCAACCTCATGTAA |
| *CSN3* | GCACTGTAGCTACTCTAGAAGATTCTCCAGAAGTTATTGAGAGCCCACCTGAGATCAACACAGTCCAA |
| *DNASE1* | CGTCACCCTCGGCCCCATCGGACGCCGTGGCTGAGATTAATTCTCTCTACGATGTCTACCCTGGGATGGCAACTTTT |
| *FOS* | TGAGTCCACACGGGGATGCTCGCTTGCAAGTCCTTGAGGCCCACAGCCTGGTGTGTTTCACGGAA |
| *GRHL1* | CCGGAGACGACTTTGTGATCCCGCCTCCTGCTAAGCTGACCCGCGGGGTTATGA |
| *HLA-DRA* | GCAGGAGATGTCTATGACTGCAAGGTGGAGCACTTGGGTTTGAATGAGCCTCTTCTCAAGCACTGGGAGTATGAAGCTCCAAA |
| *HMOX1* | GTGAGCTGACCCAAGAAGGTTTTAAGCTGGTGATGGCGTCAA |
| *IL1B* | ACAGCCATGGCACCGTACCTGAACCCATCAACGAAATGATCGGCTTACGTCACAGTGGACAGAGCACAATAGCACCCCC |
| *IL10* | AAGTCTTCAGGCCAGTAGGGGAGTTTGACATCTTCATCAACTACATAGAAACCTACATGACAACGAAGATGCAA |
| *IL10RB* | GGAGCGTATTGCTGAATGAGAGTTCAGAGTGGATAAACATCACCTTCTGTCCTGTGGATGACACCACTATCGGACCTCC |
| *IL15* | TGTACAATTGTAGCATCTTATTCAATCTATACATATGGATGCCACTTTATATACTGAAAGTGATGCTCATCCCAATTGCAAA |
| *IL18* | CATGGCACTGACCTAGCTCTCAATCATACGAAATTTGAATGACCAAGTTCTCTTCATTAACCAGGGAAATCAACCTGTAA |
| *IL1R2* | AAGGTGCCACTGATTTTTGATCCTGTCAGAAGAGAGGATTTTGGCCACCACCGGGATTAA |
| *IL1RN* | CAGTCAGTACGAACAGGGAGCAGGACAAGCGCTTTGCCTTCATCCGCTTCGACAACGGGCCCACCACCAGCTTTGAGTCAGCTGCCTGCAA |
| *IL8* | GGATACTGACTTCGATGCCAATGCATAAAAACACATTCCACACCTTTCCACCCCAAATTTATCAAAGAATTGAGAGTTATTGAGAGATGGGCCACACTGTGAAAA |
| *INSR* | AGCTGCGGTCTATCTCCGGCCTGCGTCACTTTACTGGCTATCGCATCGAGCTATCAGGCTACG |
| *IRAK-1* | GCTACGGGCATCAGTTCTTACATCAAGATAGCCCCAGCCTCATCCATGGAGATGTCAAGAGTTCCAACGTCCAA |
| *JAK1* | AGCTGTGCCTATTCGAGACCCCAAGACGGAGCAGGATGGGCACGACATTGAGAACGAGTGTCTTGGGATGGGACCAAATCATACTGTCCCTGACA |
| *LALBA* | CCAGGCCTGACAGTTACAAATGTGAGGTGTTCCGGGAGCTGAAAGACTTGAAGGGCTACGGAGGTGTCAGTTTGCCTGAATGGGTCTGTA |
| *LDLR* | CACCTTTCAGCAGCCGAGAGGGGGTAACTGGTGTGAGAGGACTGCCCTCCGCAATGGTGGCTGCCAGTACCCAATAT |
| *LYZ* | CTGGACATGGATGGATGGCTATCGAGGAGTCAGCCTGGCAAACTGGGTGTGTTTGGCCAGATGGGAAGCAATTACAACACACAAGCTACAAA |
| *MMRN2* | GACTGATAGTATGACAAGCTAAGGGCCAGCGCTTTGGGAGGCAGGCTCCCCTGTGGCCTTCTACGCCAGCCTTTTCAGAAGGATTTGGGG |
| *NENF* | GGGGTGGTGTTCGATGTCACTTCTGGAAAGGAGTTTTATGGACGAGGAGCCCCCTACAACGCCCCTGTTTCCGGAAC |
| *NR3C1* | CATGCAGAGTCATTAAGCACCCCCAGTAGAGAAGAAATCCCCAGCAGTGTGCTTGGTCGAGAGAGGGGAAATGTGATGGACTTCTATAAAACCCTAAGGGGAGGA |
| *PLAU* | GCTAGTCCAAGATCTGATGCCATTCAGCTGGGCCTGGGGAAACACAATTATTGCAGGAACCCAGACAATCA |
| *PLAUR* | TGACTGCCGCGGCCCTATGAATCAATGTCTGGAAGCGACAGGCACTAAAGGACTGAGGAACCCACACTTTGG |
| *PRKCB1* | CTGTCGTGCCAGACTCCTGAAGAAAAGACGACCAACACCATCTCCAAATTTGACAACAACGGCAACAGGGACAT |
| *SAA3* | GGATGGGGGACATTCCTCAAGGAAGCTGGTCAAGGGGCTAAAGACATGTGGAGAGCTTACCAAAA |
| *SDHD* | GATGATCGTGTCTGCGATGGACTACTCTCTGGCTGCAACCCTCACTCTTCACAGTCACTGGGGCATTGAA |
| *SELL* | ACGGGCCATGGACAATGTGTGGAAGTCATCAATAATTACACCTGCGTGCCCGTGATTTGGGGGGACCTCCGGAA |
| *SELP* | GACTGACACAAGCCACCTAGGACATACGGAGTTTTTACAAATGCTGCGTTTGACCCAAGCCCTTAAA |
| *SOCS2* | GCAACTTACATCAGCACCACCCCTGCAGCATCTCTGTAGACTCACCATTAACAAGTGTACCAGCACCGTCA |
| *STAT3* | AGCTAAATTATCAACCTTAAGATTAAAGTGTGCATTGACAAAGACTCCGGGGAA |
| *TLR2* | AGCATGGACTCACCTCCTTTAGCTGTGTCTTCATAAGCGAGACTTCATTCCTGGCAAGTGGATTATCGA |
| *TLR4* | AGACTGGTCAGGAGCTGATCTCTACAAATCCCCGACAACATCCCCATATCAACCAAGATGCTGGACCTGAGCTTTAACTAAG |
| *TNF* | CTGGTTCAACACTCAGGTCCTCTTCTCAAGCCTCAAGTAACAAGCCGGTAGCCCACGCTTGTAGCCGAAA |
| *TRAF6* | CCGTCATGCCTACGCCAGTCCCATGCACGTTCAGTGCTTTTGGTTGCCATGAAAAGATGCAGAGGAATCACTAC |

Reference List

1. Everts RE, Band MR, Liu ZL, Kumar CG, Liu L, Loor JJ, Oliveira R, Lewin HA: **A 7872 cDNA microarray and its use in bovine functional genomics**. *Vet Immunol Immunopathol* 2005, **105:**235-245.

2. Loor JJ, Everts RE, Bionaz M, Dann HM, Morin DE, Oliveira R, Rodriguez-Zas SL, Drackley JK, Lewin HA: **Nutrition-induced ketosis alters metabolic and signaling gene networks in liver of periparturient dairy cows**. *Physiol Genomics* 2007, **32:**105-116.

3. Bionaz M, Loor JJ: **Identification of reference genes for quantitative real-time PCR in the bovine mammary gland during the lactation cycle**. *Physiol Genomics* 2007, **29:**312-319.

4. Moyes KM, Drackley JK, Morin DE, Rodriguez-Zas SL, Everts RE, Lewin HA, Loor JJ: **Effect of energy balance on gene expression profiles in bovine mammary tissue [abstract]**. *FASEB J* 2008, **22:**294.4.

5. Moyes KM, Drackley JK, Morin DE, Rodriguez-Zas SL, Everts RE, Lewin HA, Loor JJ: **Effects of energy balance and *Streptococcus uberis* intramammary infection challenge on gene expression profiles in bovine mammary tissue [abstract]**. *J Dairy Sci* 2007, **90:**Suppl. 1.

6. Moyes KM, Drackley JK, Bionaz M, Morin DE, Rodriguez-Zas SL, Everts RE, Lewin HA, Loor JJ: **Gene expression networks in bovine mammary tissue during a *Streptococcus uberis* (*S. uberis*) intramammary infection challenge.** Madison, WI: National Mastitis Council; 2008:174-175.

7. Bionaz M, Loor JJ: **Gene networks driving bovine milk fat synthesis during the lactation cycle**. *BMC Genomics* 2008, **9:**366.

8. Piantoni P, Bionaz M, Graugnard DE, Daniels KM, Akers RM, Loor JJ: **Gene expression ratio stability evaluation in prepubertal bovine mammary tissue from calves fed different milk replacers reveals novel internal controls for quantitative polymerase chain reaction**. *J Nutr* 2008, **138:**1158-1164.

9. Vandesompele J, De PK, Pattyn F, Poppe B, Van RN, De PA, Speleman F: **Accurate normalization of real-time quantitative RT-PCR data by geometric averaging of multiple internal control genes**. *Genome Biol* 2002, **3:**RESEARCH0034.

10. Harvatine KJ, Bauman DE: **SREBP1 and thyroid hormone responsive spot 14 (S14) are involved in the regulation of bovine mammary lipid synthesis during diet-induced milk fat depression and treatment with CLA**. *J Nutr* 2006, **136:**2468-2474.

11. Bionaz M, Loor JJ: **ACSL1, AGPAT6, FABP3, LPIN1, and SLC27A6 are the most abundant isoforms in bovine mammary tissue and their expression is affected by stage of lactation**. *J Nutr* 2008, **138:**1019-1024.
